# A Rapid, Highly Sensitive and Open-Access SARS-CoV-2 Detection Assay for Laboratory and Home Testing

**DOI:** 10.3389/fmolb.2022.801309

**Published:** 2022-04-01

**Authors:** Max J. Kellner, James J. Ross, Jakob Schnabl, Marcus P. S. Dekens, Martin Matl, Robert Heinen, Irina Grishkovskaya, Benedikt Bauer, Johannes Stadlmann, Luis Menéndez-Arias, Andrew D. Straw, Robert Fritsche-Polanz, Marianna Traugott, Tamara Seitz, Alexander Zoufaly, Manuela Födinger, Christoph Wenisch, Johannes Zuber, Andrea Pauli, Julius Brennecke

**Affiliations:** ^1^ Research Institute of Molecular Pathology (IMP), Vienna BioCenter (VBC), Vienna, Austria; ^2^ Institute of Molecular Biotechnology of the Austrian Academy of Sciences (IMBA), Vienna BioCenter (VBC), Vienna, Austria; ^3^ Vienna BioCenter PhD Program, Doctoral School of the University of Vienna and Medical University of Vienna, Vienna, Austria; ^4^ MRC Laboratory of Molecular Biology, Cambridge, United Kingdom; ^5^ Department of Chemistry, University of Natural Resources and Life Sciences, Vienna, Austria; ^6^ Centro de Biología Molecular “Severo Ochoa” (Consejo Superior de Investigaciones Científicas and Universidad Autónoma de Madrid), Madrid, Spain; ^7^ Institute of Biology I and Bernstein Center Freiburg, Faculty of Biology, Albert-Ludwigs-University Freiburg, Freiburg, Germany; ^8^ Institute of Laboratory Diagnostics, Vienna, Austria; ^9^ 4th Medical Department with Infectious Diseases and Tropical Medicine, Vienna, Austria; ^10^ Sigmund Freud Private University, Vienna, Austria; ^11^ Medical University of Vienna, Vienna BioCenter (VBC), Vienna, Austria

**Keywords:** COVID-19 diagnostics, RT-LAMP, coronavirus, SARS-CoV-2, isothermal amplification, open-source

## Abstract

RT-qPCR-based diagnostic tests play important roles in combating virus-caused pandemics such as Covid-19. However, their dependence on sophisticated equipment and the associated costs often limits their widespread use. Loop-mediated isothermal amplification after reverse transcription (RT-LAMP) is an alternative nucleic acid detection method that overcomes these limitations. Here, we present a rapid, robust, and sensitive RT-LAMP-based SARS-CoV-2 detection assay. Our 40-min procedure bypasses the RNA isolation step, is insensitive to carryover contamination, and uses a colorimetric readout that enables robust SARS-CoV-2 detection from various sample types. Based on this assay, we have increased sensitivity and scalability by adding a nucleic acid enrichment step (Bead-LAMP), developed a version for home testing (HomeDip-LAMP), and identified open-source RT-LAMP enzymes that can be produced in any molecular biology laboratory. On a dedicated website, rtlamp.org (DOI: 10.5281/zenodo.6033689), we provide detailed protocols and videos. Our optimized, general-purpose RT-LAMP assay is an important step toward population-scale SARS-CoV-2 testing.

## Introduction

The Coronavirus Disease 2019 (COVID-19) pandemic poses unprecedented global health and economic challenges. COVID-19 is caused by infection with the single-stranded, positive-sense RNA beta-coronavirus SARS-CoV-2 (Gorbalenya et al., 2020). Despite several clinically approved options to effectively prevent and/or treat severe COVID-19, efforts to contain the spread of SARS-CoV-2 remain challenging and rely on systematic viral testing, contact tracing and isolation of infected individuals (Ferretti et al., 2020; Larremore Daniel et al., 2021). This is especially true for low-income countries, which have only limited access to COVID-19 vaccines (only 7.2% of people in low-income countries have received at least one dose at the end of 2021 (Ritchie et al., 2020)). Since SARS-CoV-2 carriers can be asymptomatic despite being infectious, a key challenge is to develop affordable and scalable technologies that enable population-wide testing (Mercer and Salit 2021; Sah et al., 2021). The gold-standard technique to detect an acute SARS-CoV-2 infection relies on nucleic acid diagnostics by RT-qPCR, which has been the method of choice due to its large dynamic range and high specificity (Corman et al., 2020). However, the need for specialized equipment and associated high cost make this technology unsuitable for low resource settings and home testing. Moreover, slow turn-around times of several hours limit the applicability of RT-qPCR-based testing for situations where rapid screening is needed (CDC 2020). Point-of-care Antigen-detecting Rapid diagnostic Tests (Ag RDTs) (“Self-Tests”) have greatly expanded population-wide surveillance of virus activity without the need of diagnostic laboratories (Brümmer et al., 2021). However, independent evaluations of commercially available Ag RDTs have shown variable and often insufficient sensitivity to detect infected individuals in the pre-symptomatic or symptomatic phase (Scheiblauer et al., 2021; Schuit et al., 2021). Moreover, Ag RDTs are single-use only devices and provide limited flexibility to 1) scale-up the number of reactions, or 2) directly modify test parameters to adapt the assay to mutated viral antigens or enable analysis of an endogenous reference protein alongside the viral antigen.

Isothermal nucleic acid amplification techniques, such as RPA (Recombinase-based Polymerase Amplification) (Piepenburg et al., 2006) or LAMP (Loop mediated isothermal amplification) (Notomi et al., 2000), have great potential to fill the technological gap required for large scale testing strategies as they enable rapid nucleic acid diagnostics with minimal equipment requirement (Niemz et al., 2011). Coupled to a reverse transcriptase step that converts viral RNA into single stranded DNA, several LAMP protocols for SARS-CoV-2 detection have been developed and applied to patient testing (Rabe and Cepko 2020; Dao Thi Viet et al., 2020; Anahtar et al., 2021; Baba et al., 2021). Innovations such as a colorimetric read-out or the combination of RT-LAMP with specific CRISPR-Cas enzymatic detection has further simplified the assay and enhanced specificity, respectively (Broughton et al., 2020; Joung et al., 2020). However, several challenges remain, especially in terms of assay robustness, compatibility with crude patient samples, limitations in sensitivity, compatibility with home testing setups, and access to the patent-protected gold-standard RT-LAMP enzymes, which poses a central bottleneck for low-income countries.

Here, we present a versatile RT-LAMP assay that overcomes the limitations of current isothermal SARS-CoV-2 detection methods. We adopted an approach to greatly reduce the risk of carry-over contamination for SARS-CoV-2 testing, increased the robustness of the assay across all tested sample types and buffer conditions by using hydroxynaphthol blue (HNB) as colorimetric readout, boosted sensitivity by at least ten-fold by combining RT-LAMP with a simple RNA enrichment procedure, benchmarked a pipette-free method that enables sensitive and specific detection of SARS-CoV-2 in home settings, and finally present a powerful RT-LAMP assay that builds exclusively on open-source enzymes.

## Results

### A Rapid, Sensitive and Specific RT-LAMP Setup for SARS-CoV-2 Detection

**FIGURE 1:**
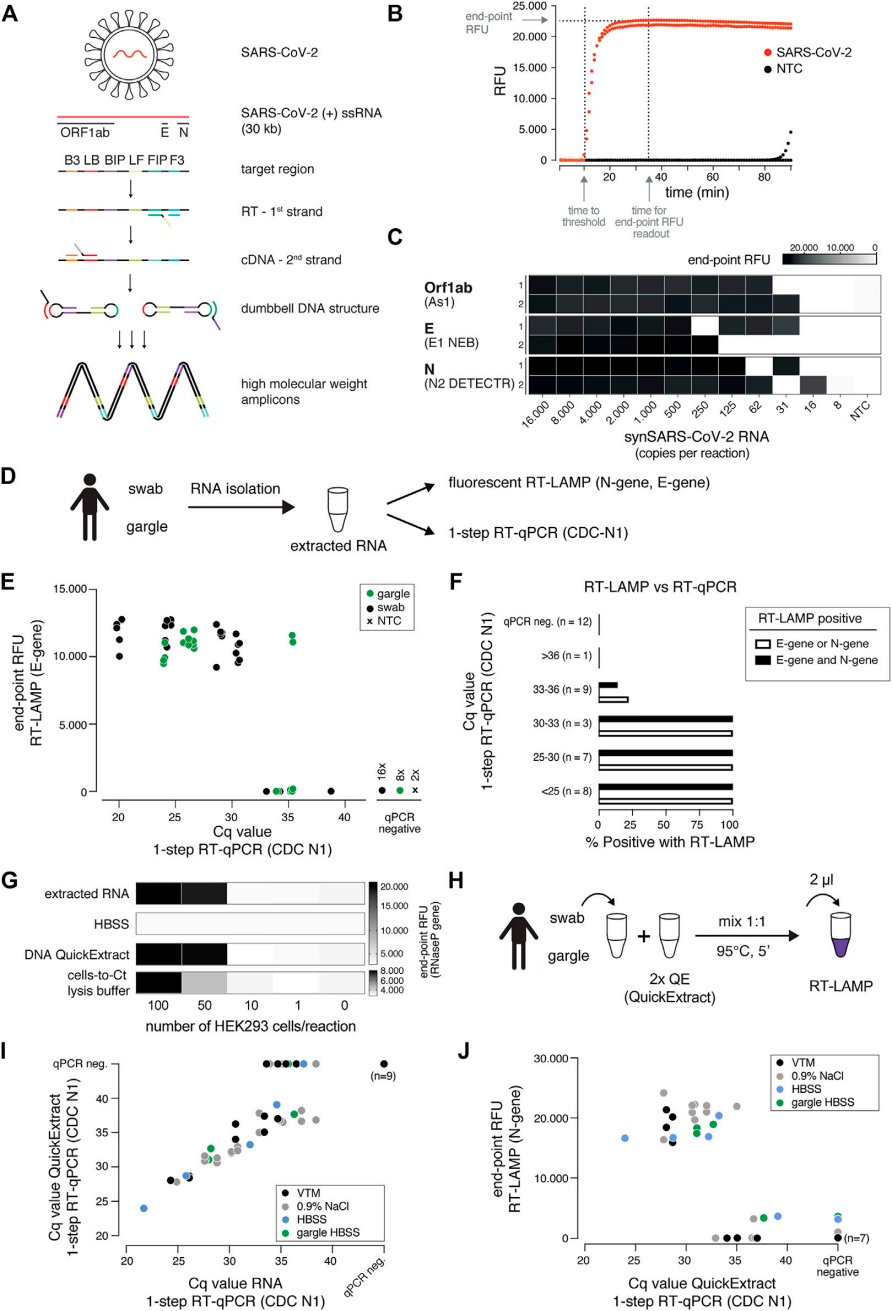
A sensitive, robust RT-LAMP assay compatible with crude patient samples. **(A)** Schematic illustrating loop-mediated amplification (LAMP) of SARS-CoV-2 RNA and the regions targeted in this study (Orf1ab, E and N genes; depicted above). Each target region is recognized by a defined set of primers (B3, LB, BIP, LF, FIP, F3). The RNA template (red) is reverse transcribed and displaced after first-strand synthesis; the outer primer binding sites are added in the subsequent amplification step. The resulting dumbbell DNA structure acts as template for further rounds of amplification, ultimately leading to high molecular weight amplicons. **(B)** Readout of a real-time fluorescence RT-LAMP reaction using 500 copies of synthetic SARS-CoV-2 (red) or water as non-targeting control (NTC, black) as input. “Time to threshold” indicates the time at which the fluorescence value reaches threshold level (equivalent to Cq value in RT-qPCR assays), “end-point RFU” indicates the fluorescence value (FAM filter set, absorption/emission at 494 nm/518 nm) after 35 min reaction time (used throughout this study unless indicated otherwise); RFU: relative fluorescence units. **(C)** Performance of the three top primer sets for RT-LAMP-based SARS-CoV-2 detection. End-point relative fluorescence units (RFUs) of RT-LAMP reactions (in duplicates) using the indicated primer sets and serially diluted synthetic SARS-CoV-2 RNA standard as input. Water was used as no-target control (NTC). **(D)** Cartoon indicating the workflow for SARS-CoV-2 detection by either RT-LAMP or 1-step RT-qPCR from patient samples (nasopharyngeal swab or gargle) with prior RNA isolation. **(E)** Comparison of RT-LAMP and RT-qPCR performance. Plotted are RT-LAMP end-point fluorescence values after 35 min versus the respective RT-qPCR Cq values. RNA was derived from gargle (green) or nasopharyngeal swabs (black); two no-target controls were included (black cross). Reactions in which no amplification was recorded are labelled as qPCR negative. **(F)** Detection rate for RT-LAMP reactions compared to a gold standard 1-step RT-qPCR assay. Shown are percentages of positive (detected in RT-LAMP and RT-qPCR) predictive agreement for sample groups (defined by RT-qPCR-derived Cq values) between RT-LAMP (using E- and/or N-gene primers) and 1-step RT-qPCR. **(G)** Performance of different crude sample preparation methods in RT-LAMP. Shown are end-point relative fluorescence units (RFUs) for RT-LAMP reactions targeting human RNAseP on sample inputs derived from defined numbers of HEK293 cells mixed 1:1 with indicated 2x buffers (extracted RNA served as a positive control). **(H)** Cartoon indicating the workflow for RT-LAMP using QuickExtract crude lysate as sample input. **(I)** Comparison of QuickExtract crude sample input versus extracted RNA as input using 1-step RT-qPCR. COVID-19 patient nasopharyngeal swabs or gargle samples (color coded according to the indicated collection medium) were either processed with the QuickExtract workflow (crude sample input) or RNA was extracted using an automated King Fisher RNA bead purification protocol. Reactions in which no amplification was recorded are labelled as qPCR negative. **(J)** Performance of RT-LAMP with QuickExtract treated crude COVID-19 patient sample input (same samples as in I). Depicted is the comparison of Cq values from RT-qPCR performed on QuickExtract treated samples versus corresponding end-point relative fluorescence units (RFUs) from RT-LAMP reactions.

SARS-CoV-2 nucleic acid diagnostic testing relies on detection of viral RNA through reverse transcription and subsequent amplification of small parts of the 30 kilobase viral genome. Considering that in SARS-CoV-2 infected human cells the various subgenomic viral RNAs are expressed at different levels (Kim et al., 2020), we benchmarked six published SARS-CoV-2 specific primer sets (see *Materials and Methods*) based on their reported high sensitivities targeting different regions of the viral genome: the 5′-located ORF1ab gene, the envelope E gene and the most 3′-located N gene encoding the nucleocapsid protein (Figure 1A) (Zhang et al., 2020a; Rabe and Cepko 2020; Broughton et al., 2020; Anahtar et al., 2021). We used RNA extracted from nasopharyngeal swabs obtained from COVID-19 patients or confirmed SARS-CoV-2 negative individuals (negative controls) and a SARS-CoV-2 RNA standard to determine primer specificity and sensitivity in RT-LAMP reactions with fluorometric real-time readout. None of the six primer sets resulted in non-specific amplification within the first 50 min in negative controls. In contrast, when using patient RNA or synthetic SARS-CoV-2 standard as input, robust target amplification occurred after 10–20 min (Figure 1B; Supplementary Figure S1). We conclude that RT-LAMP alone, without additional detection steps, is highly specific in complex human RNA samples. Throughout this study, we therefore recorded fluorescent real-time measurements or performed end-point analyses after 30–35 min reaction time.

Three primer sets enabled SARS-CoV-2 detection down to ∼30 copies per reaction (∼15 copies/µl sample input): As1, E1 (NEB) and N2 (DETECTR) targeting the Orf1ab, E- and N-gene, respectively (Figure 1C) (Rabe and Cepko 2020; Broughton et al., 2020; Zhang et al., 2020b). As previously reported (Rabe and Cepko 2020; Anahtar et al., 2021), RT-LAMP reactions with less than ∼100 target molecules exhibited stochastic on-off outcomes (Figure 1C; Supplementary Figures S1B,C). To provide estimates for the limit of detection of these primer sets, we performed RT-LAMP reactions on a synthetic SARS-CoV-2 RNA (Twist) dilution series involving twelve technical replicates of each concentration using As1 and E1 (NEB) primers. Both primer sets were able to detect reactions with 100 or more copies per reaction (RT-qPCR Cq ∼34) with >90% confidence, while maintaining 100% specificity (Supplementary Figures S1C,D). When tested with a diluted patient sample, the E1 (NEB) and N2 (DETECTR) primers performed best with a 100% cumulative detection rate of samples with RT-qPCR Cq values of less than ∼33, which corresponds to ∼100 copies of SARS-CoV-2 per reaction (Figures 1E,F; Supplementary Figure S1E).

We next compared our RT-LAMP setup with one-step RT-qPCR, using RNA isolated from nasopharyngeal swabs or gargle lavage from COVID-19 patients as input (Figures 1D–F). Using E1 or N2 primer sets for RT-LAMP, we achieved sensitive and specific detection of SARS-CoV-2 in patient samples with RT-qPCR measured Cq values of up to ∼35 (∼30 copies per reaction), independent of the patient sample type (Figure 1E). We obtained 100% positive agreement between RT-LAMP and RT-qPCR up to Cq 33 (∼100 copies per reaction) and no RT-LAMP false positives for RT-qPCR negative samples (Figure 1F). E1 and N2 primer sets performed equally well, with 100% of samples with Cq values lower than 33 being correctly identified as positive (Figure 1F). As shown recently (Zhang et al., 2020b), different primer sets could further be combined in RT-LAMP reactions in order to reduce the false negative rate caused by suboptimal sample quality or by mutations in the viral genome coinciding with primer binding sites (Artesi et al., 2020).

A major bottleneck of the predominantly used diagnostic RT-qPCR-based SARS-CoV-2 nucleic acid detection assays is their dependence on time-consuming and expensive RNA purification from patient samples. Inspired by (Ladha et al., 2020), we assessed whether direct sample input/lysis conditions are compatible with sensitive RT-LAMP. Besides simple heat inactivation, we tested two previously published lysis and sample inactivation buffers, namely DNA QuickExtract (Lucigen) (Ladha et al., 2020) and the “Cells-to-Ct” lysis buffer (Joung et al., 2017). To assess different lysis conditions, we compared crude lysates from serially diluted HEK293 cells to isolated RNA from equivalent numbers of cells as input for RT-LAMP reactions targeting the human reference gene RNaseP POP7 (Broughton et al., 2020) (Figure 1G). The direct input of heat-inactivated QuickExtract crude lysate in RT-LAMP performed equally well compared to a standard RNA extraction step in detecting RNaseP from defined numbers of cells (Figure 1G). Follow-up experiments substantiated that QuickExtract, in combination with heat treatment, effectively inactivates exogenously added RNAse A, which mimics RNase activity commonly observed in biological fluids (Bradbury 1956) (Supplementary Figure S2).

To benchmark QuickExtract solution on COVID-19 patient samples, we performed RT-qPCR on either purified patient RNA or crude QuickExtract lysate (Figure 1H). Irrespective of the sample type (swab or gargle), we observed a strong agreement between the corresponding RT-qPCR measurements (Figure 1I). Only samples with very low viral titers (high Cq values) became undetectable in the QuickExtract samples, presumably as ∼20-fold less patient material equivalent was used compared to reactions using isolated RNA as input (Figure 1I). Importantly, RT-LAMP performed equally well to extracted RNA when using QuickExtract crude sample input across different transport media and different sample types (swabs in viral transport medium (VTM), swabs in 0.9% NaCl, swabs or gargle in HBSS buffer), with a limit of RT-qPCR-measured Cq values of 33 (∼100 copies) and identical predictive performance rates (Figure 1J). No false positives were observed, demonstrating the high specificity and sensitivity of RT-LAMP on crude samples lysed and inactivated with QuickExtract solution. Heat inactivation with QuickExtract, in combination with fluorescent detection of the RT-LAMP reaction, is therefore a rapid method to detect SARS-CoV-2 in diverse patient samples.

### An Efficient Cross-Contamination Prevention System

**FIGURE 2:**
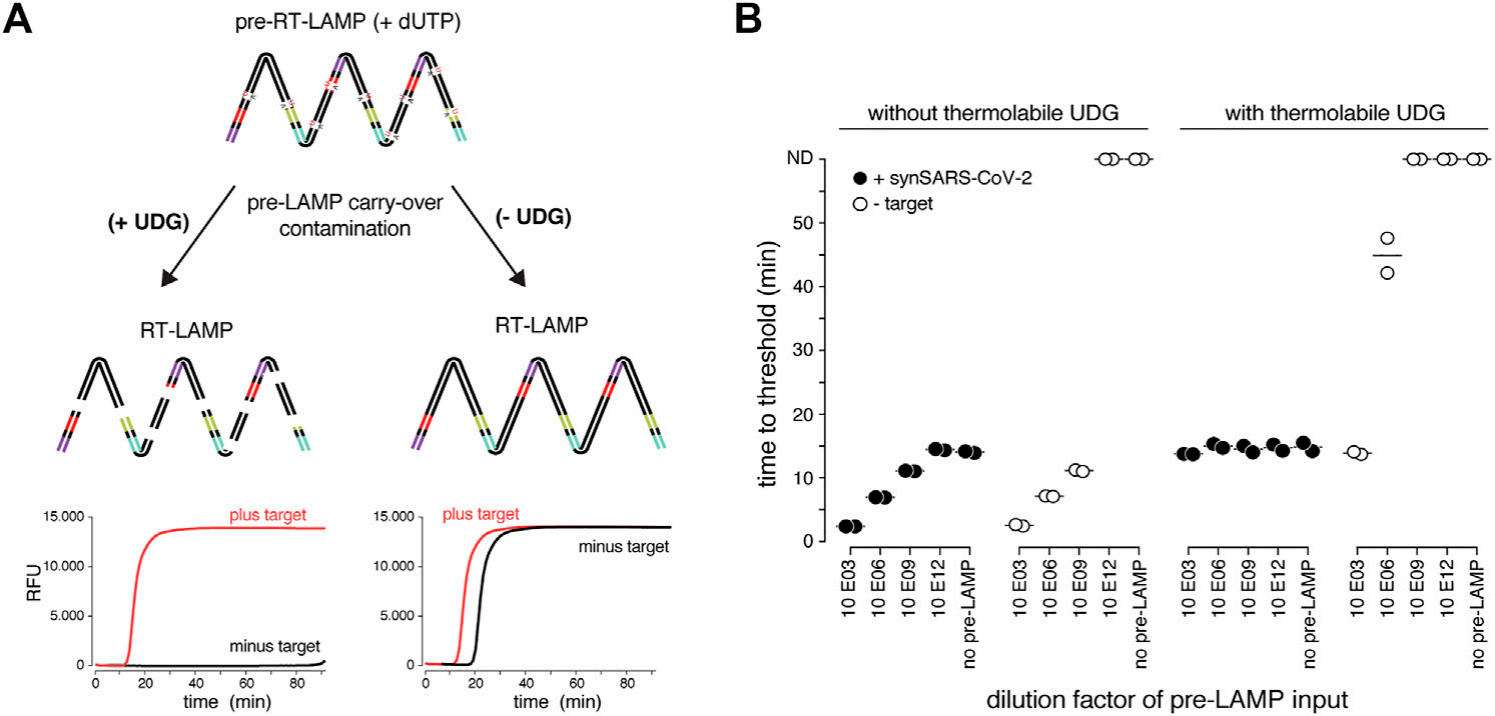
The dUTP/UDG system prevents carry-over cross-contamination in RT-LAMP. **(A)** Schematic depicting the principle of the dUTP/UDG system in preventing carry-over contamination. dUTP is incorporated into LAMP amplicons in a primary reaction (pre-RT-LAMP). dUTP containing LAMP products carried over into a subsequent reaction (RT-LAMP) are cleaved by UDG prior to LAMP-based amplification, making them unavailable as amplification templates. This allows robust discrimination between target and no-target control (left), which is challenged by cross-over contamination in the absence of UDG-mediated cleavage (right). **(B)** The dUTP/UDG system minimizes cross-over contamination. Shown are performances (time to threshold) of RT-LAMP reactions in the absence (left) or presence (right) of thermolabile UDG when using synthetic SARS-CoV-2 (filled circles) or water (open circles) as input. Reactions were supplemented with the indicated dilution of a dUTP-containing pre-LAMP reaction. All reactions were performed in duplicates.

LAMP results in the billion-fold amplification of target molecules. This poses a serious yet rarely mentioned risk, as only minor workplace or reagent contaminations with LAMP reactions will translate into large numbers of false positive assays (Robinson-McCarthy Lindsey et al., 2021; Kwok and Higuchi 1989). Inspired by previous studies, we tested whether RT-LAMP based SARS-CoV-2 detection can be combined with a contamination prevention system that utilizes dUTP and thermolabile Uracil DNA Glycosylase (UDG) (Hsieh et al., 2014; Tang et al., 2016). In this system, dUTP is incorporated into LAMP amplicons making them susceptible for uracil-base cleavage in subsequent LAMP reactions containing the UDG enzyme (Figure 2A). To mimic carry-over contaminations from amplicons of prior LAMP reactions, we supplemented pre-RT-LAMP reactions (based on the key enzymes RTx and *Bst* 2.0) with dUTP, followed by dilution and addition to reactions in the presence versus absence of thermolabile UDG (Figure 2A). Thermolabile UDG is active at room temperature yet inactivated at temperatures above 50°C. In the absence of UDG, addition of a one billion-fold diluted pre-LAMP product resulted in indistinguishable signal in target vs non-target conditions, illustrating the danger of cross-contamination. In contrast, in the presence of UDG, a 5-min pre-incubation step at room temperature reduced the amplifiable carry-over product by more than 1,000-fold, enabling specific detection in the presence of considerable cross-over contamination product (Figure 2B). We conclude that the dUTP/UDG system is compatible with RT-LAMP reactions based on *Bst* 2.0 and RTx and profoundly lowers the risk of false positives.

### A Robust Colorimetric RT-LAMP Readout Compatible With Various Input Samples

**FIGURE 3:**
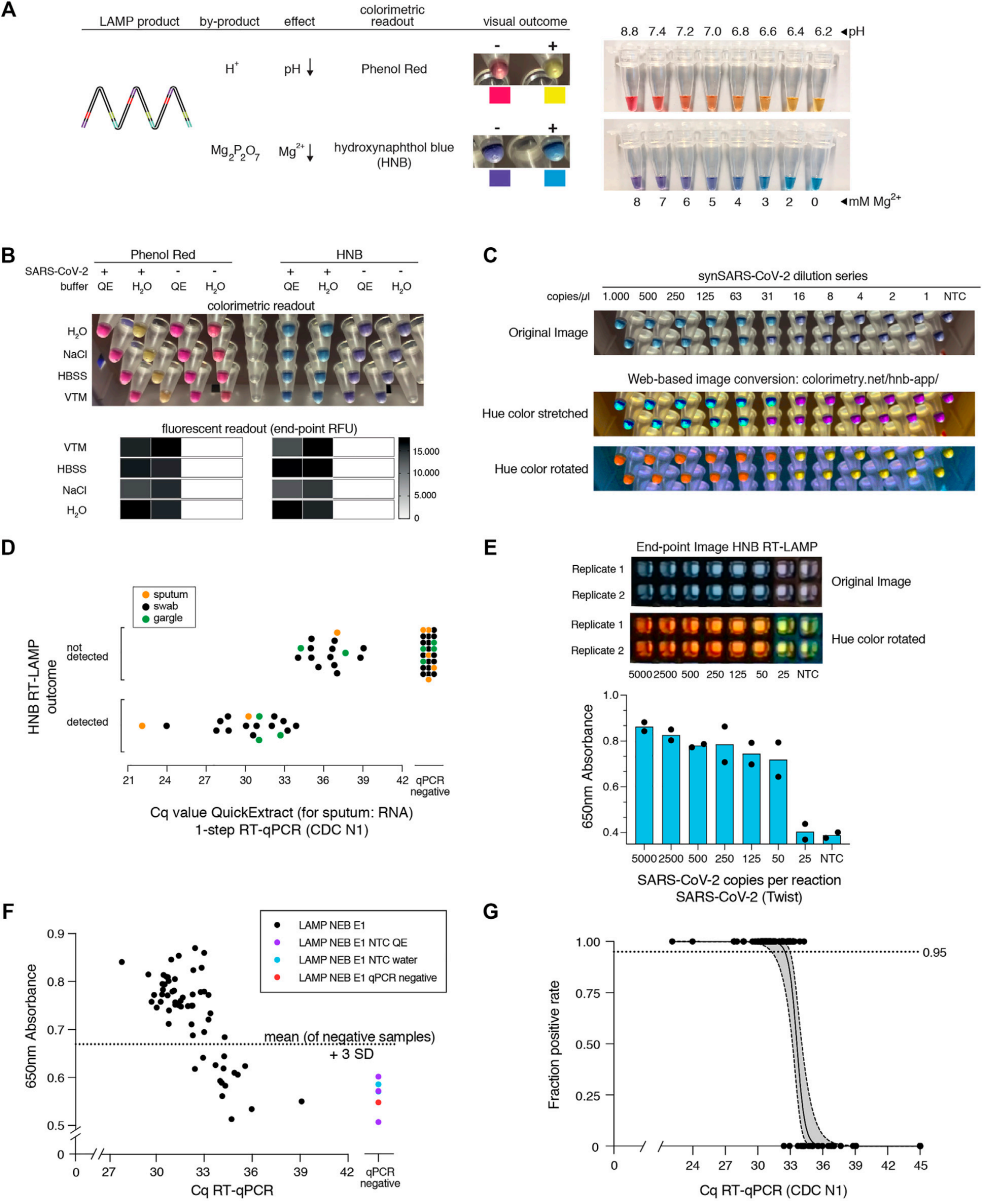
HNB RT-LAMP enables colorimetric SARS-CoV-2 detection from crude patient samples. **(A)** Schematic illustrating the properties of pH-sensitive (Phenol Red, top) and Mg^2+^ concentration sensitive (hydroxynaphthol blue, HNB, bottom) colorimetric readouts for LAMP. Phenol Red interacts with protons (H^+^) generated during DNA amplification, which causes a color change from pink/red to yellow (right: the color range of the Phenol Red-containing colorimetric RT-LAMP mastermix (NEB) at relevant pH values). Magnesium pyrophosphate precipitate produced during DNA amplification lowers the free Mg^2+^ concentration, causing a color change of the HNB dye from purple to sky-blue (right: the color range of solutions with HNB at relevant Mg^2+^ concentrations). **(B)** Influence of QuickExtract on colorimetric RT-LAMP performance using HNB or Phenol Red. Shown are RT-LAMP reaction outcomes (upper panel: colorimetric readout after 35 min, lower panel: fluorescent end-point values) when using 500 copies of synthetic SARS-CoV-2 RNA standard in indicated sample media diluted 1:1 with water or 2x QuickExtract solution as input. **(C)** Shown is the result from the QuickExtract HNB RT-LAMP dilution series, uploaded and color-converted via our custom web-based colorimetry tool. The top row shows the original image, middle and bottom rows are the color-stretched and color-rotated versions. **(D)** HNB RT-LAMP performance on COVID-19 patient samples lysed in QuickExtract solution. Shown is the binary colorimetric HNB readout of RT-LAMP reactions (N gene) using indicated patient samples (sputum (orange), swab (black), gargle (green)) plotted against the corresponding Cq values from RT-qPCR. **(E)** End-point 650 nm absorbance measurements from a HNB RT-LAMP dilution series. The raw and color-rotated images of the reactions are shown on the top, while the 650 nm absorbance measured by a Synergy H1 plate reader is shown below. **(F)** Scatter plot showing HNB RT-LAMP performance (measured by 650 nm absorbance) versus qPCR-determined Cq values on a serial dilution grid (see methods for details), including no-target controls (NTC) and a COVID-19-negative patient sample (qPCR negative). Horizontal dashed line indicates the absorbance (mean + 3SD) from five negative controls (y = 0.67). **(G)** Simple logistic regression analysis (Probit Model) of HNB RT-LAMP reaction outcomes performed on QuickExtract patient samples shown in Figure 3D and F using Graphpad Prism. Shown is the regression line representing detection limits, expressed as “Fraction positive rate”, at a given RT-qPCR Ct value with corresponding 95% confidence intervals (grey shading).

So far, we used real-time fluorescence (based on an intercalating DNA dye) to assess RT-LAMP-based target amplification. Given its dependency on specialized equipment, this detection method is prohibitive for low-resource settings or home-testing. Colorimetric detection resulting in a visual color change upon target DNA amplification provides an attractive, low-cost alternative (Goto et al., 2009; Tanner et al., 2015). Two colorimetric concepts are compatible with RT-LAMP: First, pH dependent dye indicators such as Phenol Red induce a color change from pink to yellow when the pH value of the reaction decreases upon DNA amplification (Tanner et al., 2015). Due to its pronounced color change, this is the most used readout for RT-LAMP assays. However, the pH-change dependent readout requires a weakly buffered reaction solution, which poses a great challenge when using crude sample inputs with variable pH. A second colorimetric assay utilizes metal ion indicators such as hydroxynaphthol blue (HNB), which changes color from purple to blue upon a drop in free Mg^2+^ ions, which form a Mg-pyrophosphate precipitate upon DNA amplification (Figure 3A) (Goto et al., 2009).

Colorimetric readout, either via Phenol Red or the HNB dye, can be performed simultaneously with the fluorescent readout in the same RT-LAMP reactions. When using synthetic SARS-CoV-2 standard in water as input, both colorimetric readouts mirrored the fluorescent results (Figure 3B; Supplementary Figure S3). However, when using crude QuickExtract lysate as input, the pH-dependent readout failed or was inconclusive despite successful LAMP-mediated target amplification as evidenced by the fluorescent readout (Figure 3B; Supplementary Figure S3). In contrast, the HNB-dependent color change occurred robustly in QuickExtract solution, even when mixed with various sample buffers such as VTM, NaCl or HBSS (Figure 3B; Supplementary Figure S3). We suspect that the QuickExtract solution is strongly buffered thereby preventing the pH change that is typically generated during LAMP and that is required for detection by Phenol Red. To distinguish the outcomes of HNB RT-LAMP reactions more easily, we developed a custom web-based image manipulation tool which we make freely available through https://colorimetry.net/hnb-app/ (Figure 3C). The automated image enhancement tool increases the perceptual ability to distinguish positive from negative HNB RT-LAMP reaction outcomes by 1) stretching the color of each pixel in Hue-Saturation-Lightness colorspace and increasing the saturation 4-fold, and 2) rotating the color of each pixel by 180° in Hue.

When tested in a clinical setting, RT-LAMP coupled to the HNB readout enabled detection of SARS-CoV-2 in patient samples with RT-qPCR values of up to ∼34 (corresponding to ∼50 copies per reaction of reference standard) with no false positives and 100% positive agreement up to Cq 33 (∼100 copies per reaction) (Figure 3D; Supplementary Figure S3A). The detection outcome was independent of the sample type, and we successfully used QuickExtract lysate from nasopharyngeal swabs, gargle solution or sputum samples (Figure 3D; Supplementary Figure S3B). We conclude that pH-independent dye formats, such as HNB, are superior in colorimetric RT-LAMP detection assays where strongly buffered or slightly acidic crude sample preparations are used as inputs.

To accurately determine the sensitivity threshold of HNB RT-LAMP, we generated a systematic dilution series of a positive COVID-19 patient sample in QuickExtract and used absorbance at 650 nm in a microplate reader to unambiguously determine the color change (Goto et al., 2009) (Figures 3E–G) (see *Methods*). We tested all dilutions by RT-qPCR and HNB RT-LAMP in parallel (Figure 3F). When considering samples with 650 nm absorbance values higher than for any co-measured negative control, HNB RT-LAMP allowed specific detection of samples up to Cq ∼34 with no false positives (Figure 3F). We conclude that, while read-out by fluorescence is the method of choice for high-throughput settings due to the higher dynamic range, direct absorbance measurement of the HNB-induced color change offers an attractive, alternative readout for large numbers of RT-LAMP reactions performed in parallel.

Finally, we compiled our data obtained from HNB RT-LAMP reactions on crude patient lysates and performed a simple logistic regression analysis to determine limits of detection (Figure 3G). The resulting model confirmed the high sensitivity (Ct-value at 50% detection probability: 33.61; 95% C.I at 50% detection probability: 33.25–34.0) and high specificity (100%, no false positive) of the assay with a limit of detection at 95% detection probability of roughly 100 copies per reaction (Ct-value 33) (95% C.I: 31.4–33.0) (Figure 3G). This furthermore agrees well with our estimated limit of detection determined on a synthetic standard (Supplementary Figure S1D), thus highlighting no loss in overall performance when using crude lysate in colorimetric HNB RT-LAMP reactions.

### A RT-LAMP Assay With Increased Sensitivity

**FIGURE 4:**
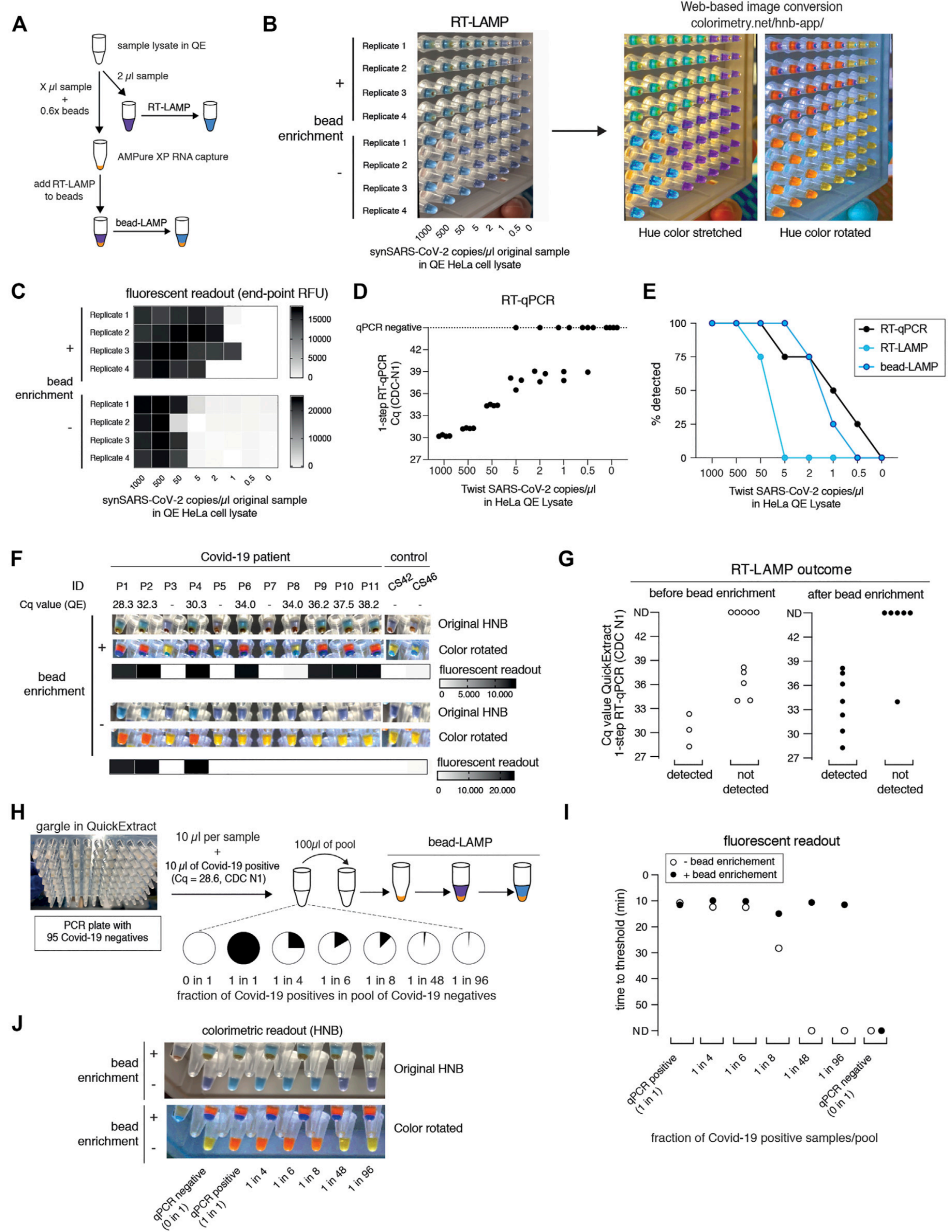
bead-LAMP increases sensitivity of RT-LAMP assays. **(A)** Schematic illustrating the bead-LAMP workflow in comparison to the regular RT-LAMP workflow. AMPure XP RNA capture beads were used at 0.6x of the volume of the sample lysate (0.6x beads). **(B)** Performance of bead-LAMP (+ bead enrichment) vs. regular RT-LAMP (− bead enrichment) using a synthetic SARS-CoV-2 RNA standard spiked-in at the indicated concentration in the original sample into HeLa cell QuickExtract (QE) lysate. 50 µl of crude sample in QE, adjusted to 100 µl final volume with 1x HBSS was used for bead-enrichment. The image shows HNB end-point colorimetric readout of bead-LAMP and RT-LAMP reactions, as well as the color-converted images produced with colorimetry.net on the right. All reactions were performed in technical quadruplicates. **(C)** End-point relative fluorescence units (RFUs), with or without prior bead enrichment for reactions shown in **(B)**. **(D)** Performance of 1-step RT-qPCR using 2 µl of the same crude sample preparations as used in **(B)** and **(C)**. **(E)** Positive detection rates of 1-step RT-qPCR, RT-LAMP and bead-LAMP for reactions shown in **(B–D)**. **(F)** Performance of bead-LAMP on a COVID-19 positive panel of patient samples in QuickExtract. The images depict the original HNB (top) and color-rotated (middle) version from colorimetric end-point readouts, and the heatmaps underneath show co-measured end-point relative fluorescence units (RFUs) of RT-LAMP reactions, with or without prior bead enrichment, using eight COVID-19-positive and five negative samples as input (P1-P11, COVID-19 patient sample; CS42 and CS46, healthy controls). 100 µl of crude sample in QE was used for bead-enrichment. Corresponding Cq values were obtained by measuring 2 µl of the same QuickExtract (QE) patient samples by 1-step RT-qPCR prior to bead enrichment. **(G)** Bead enrichment increases the sensitivity of RT-LAMP. Patient samples from **(F)** were classified as detected or not detected based on the HNB RT-LAMP assay before (left, open circles) and after (right, filled circles) bead enrichment and plotted against their respective Cq values obtained from QuickExtract (QE) RT-qPCR (Cq values for qPCR negative samples are labelled as not detected, ND). **(H)** Schematic illustrating the pooled testing strategy using bead-LAMP. A single COVID-19 positive patient gargle sample in QuickExtract (Cq ∼28; black) was mixed with different amounts of 95 pooled SARS-CoV-2 negative samples (all in QuickExtract; white) yielding seven sample pools with indicated ratios of positive to negative samples. 40–100 µl of crude sample in QE was used for bead-enrichment depending on the pool sizes. For lysate volumes smaller than 100 μl, 1x HBSS was added to obtain a final volume of 100 µl for bead-LAMP. **(I)** Shown is the performance (measured as time to threshold) of bead-LAMP (filled circles) compared to regular RT-LAMP (open circles) on the patient pools defined in **(H)**. ND = not detected within 60 min of RT-LAMP incubation. **(J)** Images showing the endpoint HNB colorimetric readout (top) and a color-rotated version underneath.

SARS-CoV-2 RT-LAMP assays are roughly ten-fold less sensitive than conventional RT-qPCR assays. When using crude samples as input (i.e. eliminating the RNA concentration step by a dedicated extraction), sensitivity is further lowered, resulting in more false negative results. To increase the sensitivity of our RT-LAMP assay, we set out to establish a simple and rapid nucleic acid enrichment step. We used carboxylated magnetic beads to concentrate RNA from QuickExtract lysates on the bead surface in the presence of crowding agents and salt (Hawkins et al., 1994). We further reasoned that, instead of eluting RNA from the beads, adding the RT-LAMP mix directly to the dry beads should increase the number of viral RNA molecules per reaction, depending on the sample input volume (Figure 4A). We tested this approach, termed bead-LAMP, by using either bead-enriched or non-enriched synthetic SARS-CoV-2 RNA in HeLa cell QuickExtract lysate as RT-LAMP input. Indeed, bead-LAMP using 50 µl QuickExtract lysate as input displayed an at least ten-fold increase in sensitivity, corresponding to a detection limit of ∼5 copies per reaction (4/4 replicates; Cq value of 37–38) (Figures 4B–D). In three out of four replicates, bead-LAMP enabled detection of as few as 2 copies/µl, and in one replicate even a single copy/µl of sample input could be detected (Figures 4B,C). Overall, bead-LAMP drastically improved performance for samples with low viral titers that were non-detectable with regular RT-LAMP (Figures 4B,C,E) and reached RT-qPCR-like sensitivity (Figure 4E). The fluorescence readout of the bead-LAMP reaction exhibited overall lower values yet similar kinetics as regular RT-LAMP (Supplementary Figure S5C), indicating that bead-LAMP is compatible with real-time kinetic analysis alongside colorimetric end-point detection (Figures 4B,C). After bead enrichment the recovery rates of synthetic SARS-CoV-2 RNA determined by RT-qPCR ranged from 68–98%, showing the high efficiency of the approach (Supplementary Figure S4).

We next tested bead-LAMP on a dilution series of a COVID-19 patient sample in QuickExtract (Cq of ∼30) and observed a similar ten-fold increased sensitivity, corresponding to a limit of detection of ∼Cq 37 in patient samples (Supplementary Figure S5A). When performing bead-LAMP on individual COVID-19 patient samples, we found a dramatic improvement in the diagnostic performance. Except for one COVID-19 positive patient that we were not able to detect via RT-LAMP for unknown reasons, all qPCR positive samples (with Cq values up to ∼38) were identified while no qPCR negative sample was detected (Figures 4F,G).

The boost in sensitivity opened the door for establishing a pooled RT-LAMP testing strategy. We mixed one crude COVID-19 positive patient gargle sample in QuickExtract (Cq ∼28) with different volumes of a pool of 95 crude SARS-CoV-2 negative gargle samples in QuickExtract (Figure 4H; Supplementary Figure S5B). Each pool was tested by standard RT-LAMP and bead-LAMP. Without bead enrichment, pools with at least 12.5% (1 out of 8) of COVID-19 positive sample were identified. In contrast, bead-LAMP enabled detection of all pools containing SARS-CoV-2, even the pool containing just 1% (1 out of 96) of COVID-19 positive sample (Figures 4I,J; Supplementary Figure S5C). An independent experiment, in which we tested bead-LAMP on a dilution series of a COVID-19 positive patient of Cq ∼30 in QuickExtract HeLa cell lysate, led to a similar conclusion: again, the pool containing only ∼1% of the COVID-19 positive sample was detectable only with prior bead enrichment (Supplementary Figures S5D–F). With merely 21 reactions (one entire 96-well plate pool, eight column pools, twelve row pools), a single positive patient of Cq ∼30 or lower can thus be theoretically detected in a total of 96 different samples (or 1 entire PCR plate). We conclude that a cheap, fast (∼5–10 min) and simple pre-enrichment step boosts the sensitivity of RT-LAMP ten- to fifty-fold, making this approach attractive for pooled testing strategies. Of note, the bead-based RNA enrichment step resulted in RT-LAMP reactions being fully compatible with the Phenol Red based colorimetric readout, even when QuickExtract lysates were used as input. While reactions without bead-enrichment failed to convert to the expected yellow color, the same input samples showed the characteristic color change when pre-purified via the bead-enrichment step (Supplementary Figure S5G).

### A Simple RT-LAMP Assay Independent of Laboratory Equipment

**FIGURE 5:**
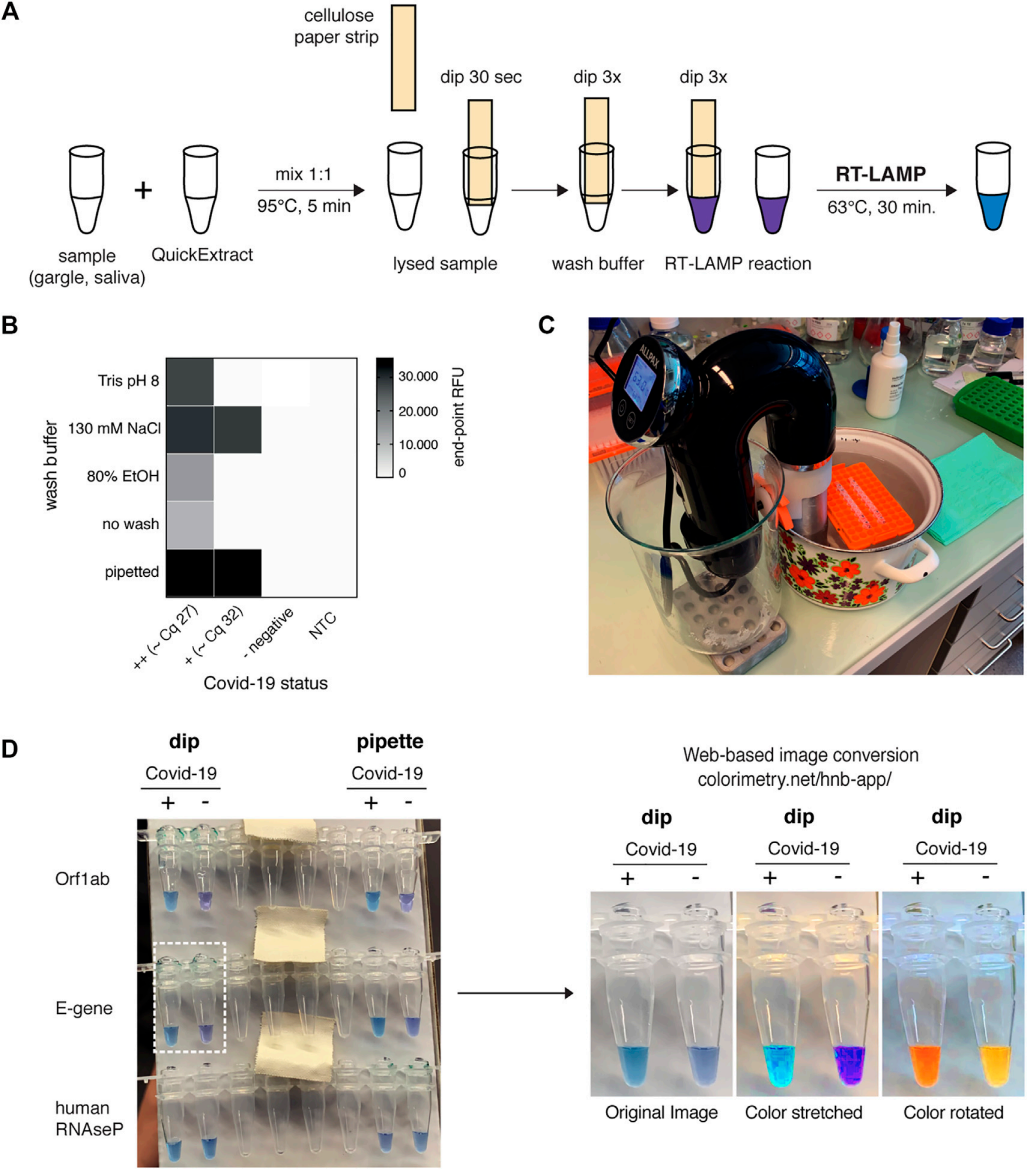
HomeDip-LAMP enables SARS-CoV-2 detection in low-resource and home settings. **(A)** Schematic depicting the HomeDip-LAMP workflow. Samples are mixed 1:1 with QuickExtract lysis buffer and inactivated at 95°C for 5 min. Cellulose paper dipsticks are loaded by dipping into the crude sample for 30 s. After a brief washing step (3x dipping into wash buffer), RNA is released into pre-distributed RT-LAMP reaction mixes by 3x dipping. RT-LAMP reactions are performed in a water bath at 63°C and read out after 35 min. **(B)** Influence of different wash conditions on SARS-CoV-2 detection using RT-LAMP with paper dipstick sample transfer. Heatmap showing end-point relative fluorescence units (RFUs) at 30 min of RT-LAMP reactions after transferring 2 µl of high titer (++, Cq ∼27), medium-to-low titer (+, Cq ∼32) or negative COVID-19 patient samples in QuickExtract into 8 µl of RT-LAMP reaction mix using cellulose paper dipsticks. Dipsticks were washed in between in indicated solutions or transferred without washing. A sample series where 2 µl were transferred by pipetting (“pipette”) is shown alongside (NTC = no target control). **(C)** Image showing the water bath setup with a Sous Vide heater (black) for HomeDip-LAMP. Reaction tubes were kept upright and submerged using floating plastic pipette tip racks (orange). **(D)** Detection of SARS-CoV-2 using HomeDip-LAMP. Left image shows true color readout (HNB dye) of HomeDip-LAMP (left 2 tubes) and pipetted LAMP (right 2 tubes) reactions using a COVID-19-positive (+) and -negative (−) patient sample in QuickExtract as input (35 min end-point; water bath incubation at 63°C). Amplicons are indicated to the left; the human RNAseP amplicon served as positive control. The images to the right show color-manipulations via the web-app (https://colorimetry.net/hnb-app/) of the two PCR tubes highlighted on the left for easier readout.

The advancements presented so far provide improved SARS-CoV-2 detection assays regarding simplicity, robustness, and sensitivity. However, our assay still required specialized laboratory equipment, such as precision pipettes or temperature-controlled incubators. We therefore explored approaches to adapt the HNB RT-LAMP protocol to home settings. QuickExtract sample inactivation can safely be done in a pot of boiling water for 5 min. In order to make RT-LAMP independent of precision pipettes, we adopted a previously reported strategy for sample clean-up and transfer using filter paper (Zou et al., 2017). In a pilot experiment, we were able to detect SARS-CoV-2 RNA from COVID-19 patients with medium viral titers (Cq ∼27) using simple Whatman filter paper dipsticks (Figures 5A,B). In addition, introducing a wash step with 130 mM sodium chloride solution increased the sensitivity and enabled SARS-CoV-2 detection in patient samples with Cq values ∼32 (∼200 copies per reaction) (Figure 5B).

Due to their isothermal nature, RT-LAMP reactions require stable incubation temperatures of ∼62–63°C. This can be provided using equipment ranging from high-end instruments to the most basal setup where boiling and room temperature water are mixed at a defined ratio and then kept insulated. We tested a commercially available sous-vide heater to create a temperature-controlled reaction environment (water bath) for home-based testing (Figure 5C). When combined with the filter paper-based sample clean-up and transfer method, this setup, termed HomeDip-LAMP, was able to accurately detect, within 35 min, two out of two viral genes in a COVID-19-positive patient gargle sample without false positives among COVID-19 negative gargle samples (Figure 5D). When compared to pipetted RT-LAMP reactions, we further observed no visual difference in final reaction color. The use of paper dipsticks instead of pipettes thus does not seem to compromise the performance of colorimetric HNB RT-LAMP reactions (Figure 5D). Taken together, our findings provide a basis for the development of a simple SARS-CoV-2 detection platform, which can be implemented in any low-tech environment.

### A Robust RT-LAMP Assay Using Open-Source Enzymes for SARS-CoV-2 Detection

A critical bottleneck in population-scale testing efforts using RT-LAMP assays, especially in low-income countries, is the dependence on an existing and robust supply chain for the two enzymes, namely the reverse transcriptase (RT) and the *Bst* DNA Polymerase. All our assays so far relied on the patent-protected enzymes RTx, a thermostable RT, and *Bst* 2.0, an engineered variant of *Bst* LF, the Large Fragment of DNA polymerase I from *Geobacillus stearothermophilis* used in original LAMP assays (Notomi et al., 2000). Given that the sequences of neither enzyme are known, we set out to identify open-source enzymes that support RT-LAMP-based SARS-CoV-2 detection without compromising assay performance.

**FIGURE 6:**
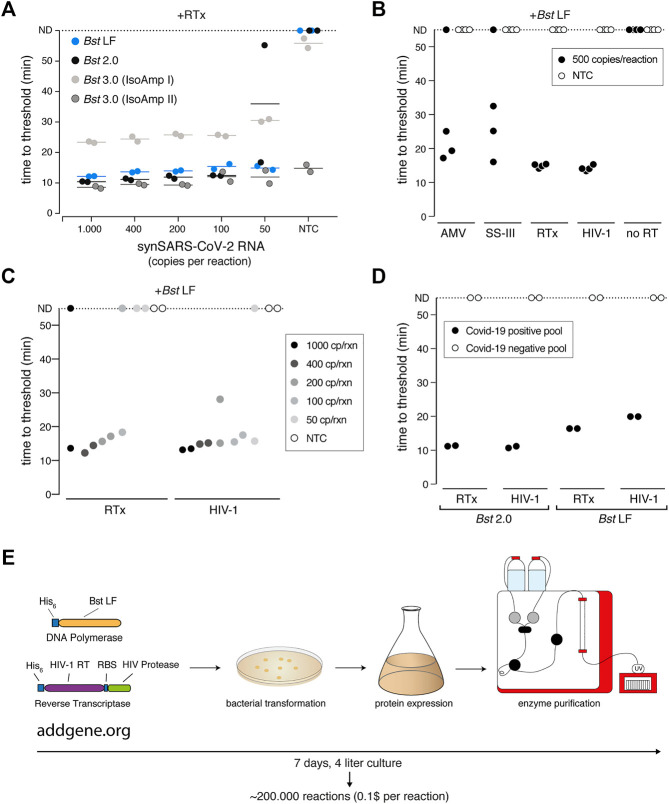
A sensitive RT-LAMP assay based on open-access enzymes. **(A)** RT-LAMP performance (measured as “time to threshold”) of different *Bst* DNA polymerase variants in combination with NEB’s RTx reverse transcriptase on synthetic SARS-CoV-2 RNA standard. For reactions in which no amplification was recorded, “time to threshold” is reported as “not detected” (ND) throughout Figures 6A–D. Reactions were performed in duplicates; water was used as no-target control (NTC). **(B)** RT-LAMP performance (measured as “time to threshold”) of different patent-protected (RTx, SuperScript III (SS-III)) and non-patent protected (AMV, HIV-1) reverse transcriptase enzymes in combination with *Bst* LF DNA polymerase on 500 copies/reaction of synthetic SARS-CoV-2 RNA standard. Reactions were performed in technical quadruplicates; water was used as no-target control (NTC) **(C)** RT-LAMP sensitivity performance (measured as “time to threshold”) of reactions containing NEB RTx or home-made HIV-1 RT, in combination with *Bst* LF DNA polymerase. Reactions contained different amounts of synthetic SARS-CoV-2 RNA standard. Reactions were performed in technical duplicates; water was used as no-target control (NTC). **(D)** Performance of RT-LAMP (measured as “time to threshold”) with different enzymatic compositions and QuickExtract patient sample as input. A pool of COVID-19 positive patient crude lysates (Pool N1-CDC, Cq 25) and a pool of COVID-19 negative crude lysates were tested in technical duplicates. **(E)** Deployment of open-access RT-LAMP. Bacterial expression plasmids can be obtained from Addgene (https://www.addgene.org/159148/, https://www.addgene.org/159149/). HIV-1 RT and Bst LF sufficient for ∼220.000 reactions of RT-LAMP can be obtained within 1 week, starting from 4 L of *E. coli* cultures.

We first focused on the *Bst* DNA Polymerase and compared the engineered *Bst* 2.0 enzyme with the wildtype *Bst* LF counterpart. *Bst* LF exhibited similar overall reaction kinetics and sensitivity compared to *Bst* 2.0 (Figure 6A). Although reported to be more salt sensitive (Maranhao et al., 2020), *Bst* LF also allowed detection of SARS-CoV-2 from crude patient lysate in QuickExtract, though it showed reduced speed in QuickExtract samples and its performance dropped more strongly for lower copy numbers in comparison to *Bst* 2.0 (Supplementary Figure S6A). An important limitation of *Bst* LF is the compromised reaction performance in the presence of dUTP, resulting in slower reaction kinetics and drop in sensitivity (Supplementary Figure S6B). Nevertheless, considering the known protein sequence of wildtype *Bst* LF (GeneBank ID: AAB52611.1) and its open-source status, *Bst* LF is the enzyme of choice for settings where engineered *Bst* variants are not available or unaffordable (Phang et al., 1995; Bhadra et al., 2021).

We next examined the reverse transcription step that is required for the robust detection of RNA targets in RT-LAMP reactions. *Bst* polymerases are reported to exhibit intrinsic RT activity (Shi et al., 2015). In particular, *Bst* 3.0 was engineered further from *Bst* 2.0 in order to display elevated intrinsic reverse transcriptase (RT) activity and increased amplification yield (Ong et al., 2015). We thus tested *Bst* LF, *Bst* 2.0 and *Bst* 3.0 in LAMP reactions lacking the dedicated reverse transcriptase RTx. *Bst* LF showed no RT activity under our assay conditions (Supplementary Figure S6C), and weak RT activity was observed for *Bst* 2.0 and *Bst* 3.0 when using universal Isothermal Amplification Buffer I (Supplementary Figure S6C). In its optimized, higher-salt buffer (Isothermal Amplification Buffer II) *Bst* 3.0 yielded strong yet non-specific amplification irrespective of the presence of a dedicated RT (Figure 6A; Supplementary Figure S6C). A CRISPR-Cas12 collateral cleavage assay (Broughton et al., 2020) on the *Bst* 3.0 LAMP products revealed that *Bst* 3.0, in the absence of a dedicated RT enzyme, led to robust amplification of the synthetic standard down to 200 copies per reaction (Supplementary Figures S6D–F). However, considering the patent-protection and the need for an additional detection step for amplicon-specific readout, we excluded *Bst* 3.0 as a possible entry point into an open-source RT-LAMP reaction. Instead, we concluded that a dedicated RT enzyme is required for efficient and specific target amplification.

To identify a thermostable, open-source RT that is active under the reaction conditions of LAMP, we first compared several RTs with the engineered RTx enzyme using synthetic SARS-CoV-2 RNA as template. We limited our test to RTs known to be active at elevated temperatures, namely AMV, Superscript III (SS-III) and a wildtype version of HIV-1 RT (Martín-Alonso et al., 2021). We found that wildtype HIV-1 RT (Álvarez et al., 2009) worked equally well in terms of efficiency, speed and sensitivity as commercial RTx (Figures 6B,C), while AMV and SuperScript-III showed only limited RT activity (Figure 6B; Supplementary Figure S7A). Moreover, wildtype HIV-1 RT in combination with *Bst* LF was fully compatible with SARS-CoV-2 detection in crude patient samples (Figure 6D). As such, RT-LAMP with HIV-1 RT and *Bst* LF was able to detect SARS-CoV-2 RNA in a pool of COVID-19 positive patients but not in a pool of COVID-19 negative lysates (Figure 6D; Supplementary Figure S7B). Reaction speed in crude patient lysates was slightly reduced compared to the gold-standard RT-LAMP reaction using RTx and *Bst* 2.0, yet initiated still within 20 min (Figure 6D; Supplementary Figure S7B). We conclude that the combination of wildtype HIV-1 RT and *Bst* LF are fully able to perform RT-LAMP under our optimized reaction conditions with crude patient samples as input. These findings open the door for any laboratory to establish their own, home-made RT-LAMP reaction mix to enable SARS-CoV-2 and other pathogen testing (Figure 6E).

## Discussion

RT-LAMP is an inexpensive and specific nucleic acid detection assay that provides test results in about 30 minutes. Its independence from specialized laboratory equipment and its compatibility with crude patient samples as well as colorimetric visual readout make it highly attractive for settings with limited resources or for population-scale testing. In this study, we systematically optimized every step of the RT-LAMP assay for crude sample input to make it more sensitive, more robust, and simpler. In order to make RT-LAMP more accessible and to disseminate the improved protocols, we built a website (www.rtlamp.org; zipped web archive can be downloaded at DOI: 10.5281/zenodo.6033689) where background information, protocols, training videos and updates of our newly established RT-LAMP assays are provided.

Most SARS-CoV-2 diagnostic assays include an expensive and lengthy RNA isolation step. To circumvent this problem, several crude sample inactivation protocols have been developed that are compatible with direct downstream reverse transcription and amplification steps (Myhrvold et al., 2018; Rabe and Cepko 2020; Ladha et al., 2020). While these advancements have simplified SARS-CoV-2 detection considerably, the use of crude lysates as input for direct RT-LAMP reactions with the most used pH-indicator-based colorimetric readout face several challenges. For example, strongly buffered lysis solutions such as QuickExtract are not compatible with Phenol Red dye detection, resulting in substantial false negative rates. Similarly, false positives have been reported for patient sample types with acidic pH such as saliva (Uribe-Alvarez et al., 2021). We employed the known metal indicator hydroxynaphthol blue (HNB) as a robust alternative for colorimetric detection of SARS-CoV-2 with no false positives and detection rates identical to highly sensitive fluorescent LAMP assays for any tested sample buffer (Figure 3; Supplementary Figure S3). HNB RT-LAMP, particularly when employed in combination with the dUTP/UDG cross-contamination prevention system (Figure 2), is therefore a robust and streamlined assay suited for SARS-CoV-2 testing in home settings.

A major drawback of using patient samples directly for nucleic acid detection is the resulting drop in sensitivity. While an upstream RNA isolation step allows the concentration of viral template molecules, this is not the case for crude extraction methods. With a robust limit of detection of ∼50–100 copies per reaction (Figures 1C, 3G; Supplementary Figure S1D), RT-LAMP with crude patient sample input can only detect medium to high viral titers. Nevertheless, this level of sensitivity is only achieved by a small fraction of commercially available SARS-CoV-2 Antigen-detecting Rapid diagnostic Tests (6 out of 122 evaluated tests showed a positive detected rate above 90% at this viral load (Scheiblauer et al., 2021)).

Our development of bead-LAMP, a simple RNA enrichment protocol with magnetic beads, sets the stage for highly sensitive SARS-CoV-2 detection in samples from individual patients or patient pools (Figure 4). While similar to a recently reported protocol based on silica particles (Rabe and Cepko 2020), our approach requires only a magnet and adds just 5–10 min to the standard protocol. Bead-LAMP does not require centrifugation and can be performed manually with a simple magnet, an automated magnetic particle processor like the KingFisher (Thermo Scientific) or on fully automated liquid handling platforms. It is especially suited for mass-scale pathogen surveillance via sample pooling strategies. Combined with the HNB colorimetric read-out, bead-LAMP allows for screening hundreds of individuals in pooled reactions in simple PCR strips. Bead-LAMP is also an attractive alternative to ultra-sensitive RT-qPCR when used on single patient samples (Figures 4F,G).

Overall, we conclude that based on the cumulative data presented in this paper, both RT-LAMP assays are of sufficient sensitivity to detect all samples with infectious viral loads (Wölfel et al., 2020; Jones Terry et al., 2021), highlighting the relevance of RT-LAMP for population screening.

While bead-LAMP enables pooled testing, reliable and sensitive home-tests provide important alternative strategies in combating the COVID-19 pandemic (Taipale et al., 2020). Towards this end, we present a simple strategy for sample RNA binding and transfer using cellulose paper strips. With HomeDip-LAMP, SARS-CoV-2 detection can be performed in home settings without the use of precision pipettes (Figure 5). Only sample inactivation buffer, paper-strips, wash and reaction solution together with a stable heat-source such as a water bath are required. We envision that a combination of bead-LAMP with HomeDip-LAMP could be adapted for sensitive home testing. In such a combined approach, beads could be added to the inactivated sample, followed by binding to a magnetic rod and dipping as described for cellulose paper strips.

Establishing RT-LAMP based SARS-CoV-2 testing in developing countries is severely hampered by unreliable or non-existing supply chains. The gold-standard RT-LAMP enzymes *Bst* 2.0 and RTx are engineered and proprietary enzymes, making their on-site production impossible. In our tests, the wildtype *Bst* LF enzyme performed equally well to *Bst* 2.0, with the exception of dUTP incorporation. One of our most significant findings was the identification of a wildtype HIV-1 RT enzyme as a comparable alternative to the RTx enzyme. *Bst* LF and HIV-1 RT can be recombinantly produced at high yields using simple molecular biology equipment (Boretto et al., 2001). The implications of our identification of open-access enzymes that support rapid and sensitive RT-LAMP are profound: in principle, any molecular biology lab with expertise in protein purification will be able to generate a robust and sensitive in-house RT-LAMP reaction mix. Moreover, several innovative strategies for enzyme production (cellular reagents (Bhadra et al., 2018)) and purification (“teabag” (Castaldo et al., 2016)) have been developed for low-resource environments.

In summary, our improvements over existing RT-LAMP workflows enable the robust, inexpensive and ultra-sensitive detection of SARS-CoV-2. Our findings provide the basis for future clinical performance studies with the ultimate goal to make “testing for everyone” a reality. Combating the COVID-19 pandemic will require access to diagnostic tests in all countries (Mercer and Salit 2021). We hope that the establishment of an RT-LAMP assay using only open-access enzymes will be an important step forward to meet precisely this need.

## Materials and Methods

### Clinical Sample Collection and Ethics

The present study includes preliminary investigations and results approved by the local Ethic Committee of Vienna (#EK 20-208-0920). A total of 85 respiratory samples were used to validate our assay, including 65 naso/oro-pharyngeal swabs, 12 gargle lavages and 8 sputum samples. Sample type and collection media are indicated in the Figures and respective Figure legends. For sample collection, handling and processing, we strictly adhered to the Biosafety protocols implemented at the Institutes and approved for the work with specimen containing SARS-CoV-2. This includes wearing appropriate personal protective gear during collection and handling of samples, as well as working in a BSL-2 biosafety cabinet prior to virus inactivation. Nasopharyngeal swabs were collected in 1.5–3 ml VTM, 0.9% NaCl solution or 1x HBSS (Gibco: 140 mM NaCl, 5 mM KCl, 1 mM CaCl_2_, 0.4 mM MgSO_4_-7H_2_O, 0.5 mM MgCl_2_-6H_2_O, 0.3 mM Na_2_HPO_4_-2H_2_O, 0.4 mM KH_2_PO_4_, 6 mM d-Glucose, 4 mM NaHCO_3_). Gargle samples were collected from swab-matched patients by letting individuals gargle for 1 min with 10 ml of HBSS or 0.9% Saline solution. Sputum samples were prepared by mixing sputum material 1:1 with 2x Sputolysin solution (6.5 mM DTT in HBSS) and incubation at room temperature for 15 min.

### RNA Extraction From Patient Material

Total RNA was isolated from 100 µl of nasopharyngeal swabs or cell-enriched gargling solution using a lysis step based on guanidine thiocyanate (adapted from Boom et al., 1990) and 20 µl of carboxylated magnetic beads (GE Healthcare, CAT:65152105050450) applied in 400 µl of Ethanol on the magnetic particle processor KingFisher (Thermo). After a 5-min incubation at room temperature, DNA was digested with DNaseI for 15 min at 37°C, followed by a series of wash steps. RNA was eluted from the beads in 50 µl RNase free H_2_O for 5 min at 60°C.

### Crude Sample Inactivation Using QuickExtract DNA Solution

50 µl of nasopharyngeal swabs, gargle solution or sputum sample were mixed 1:1 with 2x QuickExtract DNA extraction solution (Lucigen) and heat inactivated for 5 min at 95°C. Samples were then stored on ice until further use or frozen at −80°C.

### Preparation of a Sample Dilution Grid

To prepare an 8 × 8 sample dilution grid (used in Figure 3F), a quantified RT-qPCR SARS-CoV-2 positive sample was first diluted horizontally in the first row and vertically in the first column. Equal amounts from column 1/rows 2 to 8, were then distributed over columns 2 to 8, followed by mixing equal amounts of sample diluted in row 1/columns 2 to 8 with distributed samples in rows 2 to 8 to generate a sample dilution grid. This means each column and row, except the well in row 1/column 1, was mixed with each other, generating all possible sample dilution combinations. The resulting sample dilution grid was then measured via RT-qPCR to determine sample Cq values.

### RT-qPCR

For detecting the viral N-gene via RT-qPCR, 1-step RT-qPCR was performed using the SuperScript III Platinum One-Step qRT-PCR Kit (Thermofisher) or Luna Universal One-Step RT-qPCR Kit (NEB) and 1.5 µl of reference primer/probe sets CDC-N1 (IDT 10006713) or CDC-N2 (IDT 10006713) per 20 µl reaction. Reactions were run at 55°C for 15 min, 95°C for 2 min, followed by 45 cycles of 95°C for 10 s and 55°C for 45 s in a BioRad CFX qPCR cycler. Each RT-qPCR reaction contained either 5 µl (N-gene, extracted RNA) or 2 µl (N-gene, QuickExtract lysate) of sample input per 20 µl reaction.

### Fluorescent RT-LAMP

Fluorescent RT-LAMP reactions were set up using the NEB Warmstart RT-LAMP kit or individual enzymes. For reactions using the RT-LAMP kit, Warmstart RT-LAMP master mix (1x final, 2x stock) was mixed with primer solution (1x final, 10x stock) containing all six LAMP primers (B3, F3, LB, LB, FIP, BIP), LAMP dye (1x final, 50x stock) or Syto9 (1 µM final, 50 µM stock), sample and nuclease-free water. Primers were used at final concentrations of 0.2 µM for F3/B3, 0.4 µM for LB/LF (except for N2 DETECTR, LB/LF 0.8 µM) and 1.6 µM FIP and BIP. Typical final reaction volumes were 10 µl or 20 µl containing 2 µl of sample.

For LAMP reactions using individual polymerases, RT-LAMP reactions were set up using NEB 1x Isothermal Amplification Buffer (*Bst* LF, *Bst* 2.0, *Bst* 3.0) or NEB 1x Isothermal Amplification Buffer II (*Bst* 3.0), 6 mM MgSO4 (8 mM final; 2 mM MgSO_4_ are present in Isothermal Buffer I), 0.3 U/µl NEB Warmstart RTx, 0.32 U/µl NEB *Bst* DNA polymerase (LF, 2.0 or 3.0), 1.4 mM of each dNTP (Larova, 25 mM of each dNTP stock solution), 1x fluorescent dye or 1 µM Syto9, sample and nuclease-free water.

For LAMP reactions testing individual RT-enzymes, RT-LAMP reactions were set up using NEB 1x Isothermal Amplification Buffer (*Bst* LF, *Bst* 2.0, *Bst* 3.0) or NEB 1x Isothermal Amplification Buffer II (*Bst* 3.0), 6 mM MgSO4 (8 mM final; 2 mM MgSO_4_ are present in Isothermal Buffer I), 1.4 mM of each dNTP (Larova, 25 mM of each dNTP stock solution), 0.32 U/µl NEB *Bst* DNA polymerase (LF, 2.0 or 3.0), 0.3 U/µl, Warmstart RTx (NEB), 0.2 U/µl AMV RT (NEB), 4 U/µl SuperScript III (Thermofisher), 50 nM of home-made HIV-1 RT (BH10) diluted in 1x dilution buffer (TrisHCl pH 7.5, 50 mM NaCl, 0.5 mM TCEP) and 1x fluorescent dye or 1 µM Syto9, sample and nuclease-free water.

Reactions were run at 63°C (62°C for N2 DETECTR and primer comparison) for 30–60 min in a BioRad CFX Connect qPCR cycler with SYBR readings every minute.

### Direct Sample Lysis Buffer Test

HEK293 cells were trypsinized and counted to make the appropriate dilutions in HBSS. The dilutions were mixed 1:1 with respective lysis buffers and treated as follows: Cells for no extraction were incubated for 5 min at room temperature. QuickExtract samples were incubated at 95°C for 5 min. Cells lysed in the home-made buffer (19.2 mM Tris–HCl (pH 7.8), 1 mM MgCl_2_, 0.88 mM CaCl_2_, 20 μM DTT, 2% (wt/vol) Triton X-100) were incubated for 5 min at room temperature before incubation at 95°C for 5 min. For extracted RNA, RNA was purified from 1e5 HEK293 cells using standard Trizol RNA extraction and diluted to cell/reaction equivalents.

### dUTP/UDG Contamination Prevention System

Reactions were set up to contain NEB 1x Isothermal Amplification Buffer, 1.4 mM of each dATP, dCTP, dGTP, 0.7 mM dUTP, 0.7 mM dTTP, 6 mM MgSO4 (100 mM stock, NEB), 0.32 U/µl NEB *Bst* 2.0 polymerase, 0.3 U/µl NEB Warmstart RTx Reverse Transcriptase, 0.02 U/µl NEB Antarctic thermolabile UDG, sample and nuclease-free water. Reactions were set up on ice and incubated at room temperature for 5 min before being transferred to 63°C to start RT-LAMP reactions under standard conditions described above. For demonstrating carry-over contamination, reactions either contained UDG (+UDG) or water (−UDG) and different amounts of pre-amplified RT-LAMP product (pre-RT-LAMP). Pre-RT-LAMP reactions were performed with dUTP, E-gene primer and 500 copies of Twist synthetic RNA standard for 60 min at 63°C. Serial dilutions were made by mixing 1 µl of dUTP-containing pre-RT-LAMP product with 999 µl of nuclease-free water to get 1e3-, 1e6-, 1e9- and 1e12-fold dilutions of pre-RT-LAMP, followed by addition of 2 µl diluted pre-RT-LAMP product to dUTP/UDG RT-LAMP reactions.

### Colorimetric LAMP

For HNB colorimetric RT-LAMP detection, reactions were set up as in fluorescent RT-LAMP with the addition of 120 µM HNB dye solution (20 mM stock in nuclease-free water). Phenol Red colorimetric reactions were performed using the NEB WarmStart colorimetric LAMP 2x master mix and the same final primer concentrations as in fluorescent RT-LAMP reactions. HNB and Phenol colorimetric reactions further contained 1x fluorescent LAMP dye (50x stock from LAMP kit) or 1 µM Syto9 dye (50 µM Stock) to measure fluorescence in parallel.

### HNB RT-LAMP Absorbance Measurements

For the absorbance measurements of HNB RT-LAMP reactions shown in Figure 3, HNB RT-LAMP reactions were set up as described above and transferred to 384-well plate low volume microtiter plates (Corning). The plate was then incubated at 63°C for 35 min in a Hybex incubator (Hybex 1057-30, SciGene) equipped with a heated lid. The plate was subsequently transferred to a Synergy H1 microplate reader where the 650 nm absorbance of each reaction was measured.

### Bead-LAMP

For bead enrichment, variable volumes of sample in QuickExtract (40 µl up to 100 µl) were adjusted to a final volume of 100 µl with HBSS, mixed with 0.6x of beads (1:5 dilution of Agencourt RNAClean XP in 2.5 M NaCl, 10 mM Tris-HCl pH 8.0, 20% (w/v) PEG 8000, 0.05% Tween 20, 5 mM NaN_3_) and incubated for 5 min at room temperature. Beads were captured with a magnet for 5 min and then washed twice with 85% ethanol for 30 s. The beads were air dried for 5 min and then eluted directly in 20 µl colorimetric HNB LAMP reaction mix containing 1x NEB WarmStart LAMP kit, 1x Fluorescent LAMP dye, 120 µM HNB dye solution and 1x primer mix. No additional volume for dry beads was factored into the RT-LAMP reaction mix such that reactions were completed with nuclease free water to have final reaction volumes of 20 µl.

As sample input for pooled bead-LAMP (Figures 4H−J; Supplementary Figures S5B−D), sample pools were prepared by mixing 10 µl of a COVID-19 positive patient gargle sample in QuickExtract with different amounts of a COVID-19 negative gargle sample pool (*n* = 95) in QuickExtract (10 µl per sample). For pool volumes <100 μl, the volume was filled up to 100 µl with HBSS:QuickExtract (1:1); for pool volumes >100 μl, an aliquot of 100 µl were taken out after pooling for subsequent RT-LAMP or bead-LAMP. 40 µl (matching the smallest pooled sample volume) of a COVID-19 positive or negative patient gargle sample were used as positive (qPCR positive) or negative (qPCR negative) controls, and also filled up to 100 µl with HBSS:QuickExtract (1:1) before LAMP.

As sample input for the proof-of-concept experiment shown in Supplementary Figures S5D–F, sample pools containing different numbers of COVID-19 positive patient gargle sample in QuickExtract were mixed with HeLa cell lysate in QuickExtract. The HeLa cell lysate was prepared by adding 500 µl of HBSS and 500 µl of 2x QuickExtract solution to a HeLa cell pellet containing one million cells, followed by cell lysis for 5 min at 95°C. The stock lysate of 1,000 cells/µl was then diluted in 1x heat inactivated QuickExtract buffer (diluted to 1x in HBSS) to a final concentration of 20 cells/µl. This concentration was chosen as QuickExtract lysate from gargle or swabs roughly yields 200 pg/μl of RNA or 20 cells/µl. This COVID-19 negative QuickExtract lysate was used to spike-in various amounts of COVID-19 positive patient QuickExtract lysate.

Bead-LAMP using Phenol Red as colorimetric read-out (Supplementary Figure S5G) was performed with WarmStart colorimetric LAMP 2x master mix (NEB) instead of the HNB containing RT-LAMP mix.

### Assessment of Bead Enrichment

For evaluation of the recovery rate after bead enrichment different dilutions of Twist synthetic SARS-CoV-2 RNA standard were made in HeLa cell lysate. 40 µl of sample was adjusted to 100 µl with QuickExtract diluted 1:1 with HBSS. Bead enrichment was performed as described for bead-LAMP. Nucleic acids were eluted with 20 µl nuclease-free water for 5 min at 63°C. SARS-CoV-2 RNA concentrations were determined in the input (before enrichment) and eluate (after bead enrichment) by RT-qPCR.

### HomeDip-LAMP

Reactions for HomeDip-LAMP were set up as for HNB colorimetric LAMP, with final reaction volumes (excluding sample volume) being 25 µl. Filter paper dipsticks (dimensions: 2 × 10 mm) were cut from filter paper (Fisher Scientific, cat. number 09-790-14D). Using forceps, dipsticks were dipped into 2 µl of patient sample for 30 s, allowing the liquid to be drawn entirely onto the paper. The paper strips were then washed by rapidly submerging (“dipping”) three times into wash solution, typically 130 mM NaCl. Sample strips were then dipped three times into the PCR tubes containing 25 µl of pre-distributed HNB RT-LAMP reaction mixes. The RT-LAMP reaction was performed for 35 min in a water bath that was temperature-controlled by a sous-vide heater (Allpax) set to 63°C. PCR tubes were kept upright and submerged during incubation by floating pipette tip racks.

### Preparation of crRNAs for Cas12 Detection

LbaCas12a guide RNAs were ordered as reverse complementary Ultramers from IDT. A T7-3G minimal promoter sequence was added for T7 *in vitro* transcription. 1 µM Ultramer was annealed with 1 µM T7-3G oligonucleotide in 1x Taq Buffer (NEB) in a final volume of 10 µl by heating the reaction up to 95°C for 5 min, followed by slowly cooling down to 4°C with a 0.8°C/s ramp rate. One microliter of 1:10-diluted annealing reaction was used for T7 *in vitro* transcription using the Invitrogen MEGAScript T7 Transcription kit following the manufacturer instruction. RNA was transcribed for 16 h at 37°C and purified using AmpureXP RNA beads following instructions described in (Kellner et al., 2019).

### Cas12-Detection of RT-LAMP Product

RT-LAMP was set-up as described above and run at 62°C for 60 min. Meanwhile, 50 nM purified crRNA was mixed with 62.5 nM EnGen LbCas12 (NEB) in 1x NEB Buffer 2.1 and a final volume of 20 µl. The RNP complex was then incubated for 30 min in a heat-block and kept on ice until use. For detection, 2 µl of the RT-LAMP product and 125 nM ssDNA sensor (Invitrogen, DNaseAlert HEX fluorophor) were added to 20 µl of the Cas12-RNP complex on ice. Reporter cleavage was monitored in real-time using a BioRad QFX qPCR cycler with measurements taken every 5 min for a total of 60 min.

### Expression and Purification of HIV-1 RT

Recombinant heterodimeric HIV-1 RT (strain BH10, GenBank accession number AH002345) was expressed and purified using a modified version of plasmid p66RTB, as previously described (Boretto et al., 2001; Matamoros et al., 2005). HIV-1 RT p66 subunits carrying a His_6_ tag at their C-terminus were co-expressed with the HIV-1 protease using the *Escherichia coli* XL1 Blue strain. The resulting p66/p51 heterodimers were purified to homogeneity by ionic exchange on cellulose phosphate P11 (Whatman), followed by affinity chromatography on Ni^2+^–nitriloacetic–agarose (ProBond™ resin, Invitrogen). HIV-1 RT-containing fractions were pooled and dialyzed against 50 mM Tris-HCl pH 7.0 buffer, containing 25 mM NaCl, 1 mM EDTA, 10% (w/v) glycerol, and 1 mM DTT. After dialysis, enzymes were concentrated by centrifugation in Centriprep® 30K and Amicon^®^ Ultra-4 Ultracel®-10K devices (Merck Millipore Ltd.). Purity of enzymes was assessed by SDS–polyacrylamide gel electrophoresis. RT concentrations were determined spectrophotometrically by assuming a molar extinction coefficient of 2.6 × 10^5^ M^−1^ cm^−1^ at 280 nm. RT active-site titration was carried out as previously described (Kati et al., 1992; Menéndez-Arias 1998).

## Primer sequences

xxx

**Table:** 

Primer sequences for RT-LAMP		
Name	Sequence	Reference
DETECTR N-gene F3	AACACAAGCTTTCGGCAG	Broughton et al. (2020)
DETECTR N-gene B3	GAA​ATT​TGG​ATC​TTT​GTC​ATC​C	
DETECTR N-gene FIP	TGC​GGC​CAA​TGT​TTG​TAA​TCA​GCC​AAG​GAA​ATT​TTG​GGG​AC	
DETECTR N-gene BIP	CGC​ATT​GGC​ATG​GAA​GTC​ACT​TTG​ATG​GCA​CCT​GTG​TAG	
DETECTR N-gene LF	TTC​CTT​GTC​TGA​TTA​GTT​C	
DETECTR N-gene LB	ACCTTCGGGAACGTGGTT	
NEB E1-F3	TGA​GTA​CGA​ACT​TAT​GTA​CTC​AT	Zhang et al. (2020a)
NEB E1-B3	TTC​AGA​TTT​TTA​ACA​CGA​GAG​T	
NEB E1-FIP	ACC​ACG​AAA​GCA​AGA​AAA​AGA​AGT​TCG​TTT​CGG​AAG​AGA​CAG	
NEB E1-BIP	TTG​CTA​GTT​ACA​CTA​GCC​ATC​CTT​AGG​TTT​TAC​AAG​ACT​CAC​GT	
NEB E1-LB	GCG​CTT​CGA​TTG​TGT​GCG​T	
NEB E1-LF	CGCTATTAACTATTAACG	
As1_F3	CGGTGGACAAATTGTCAC	Rabe and Cepko (2020)
As1_B3	CTT​CTC​TGG​ATT​TAA​CAC​ACT​T	
As1_LF	TTA​CAA​GCT​TAA​AGA​ATG​TCT​GAA​CAC​T	
As1_LB	TTG​AAT​TTA​GGT​GAA​ACA​TTT​GTC​ACG	
As1_FIP	TCA​GCA​CAC​AAA​GCC​AAA​AAT​TTA​TCT​GTG​CAA​AGG​AAA​TTA​AGG​AG	
As1_BIP	TAT​TGG​TGG​AGC​TAA​ACT​TAA​AGC​CCT​GTA​CAA​TCC​CTT​TGA​GTG	
As1e_FIP	TCA​GCA​CAC​AAA​GCC​AAA​AAT​TTA​TTT​TTC​TGT​GCA​AAG​GAA​ATT​AAG​GAG	
As1e_BIP	TAT​TGG​TGG​AGC​TAA​ACT​TAA​AGC​CTT​TTC​TGT​ACA​ATC​CCT​TTG​AGT​G	
NEB N-gene-A-F3	TGG​CTA​CTA​CCG​AAG​AGC​T	Zhang et al. (2020b)
NEB N-gene-A-B3	TGC​AGC​ATT​GTT​AGC​AGG​AT	
NEB N-gene-A-FIP	TCT​GGC​CCA​GTT​CCT​AGG​TAG​TCC​AGA​CGA​ATT​CGT​GGT​GG	
NEB N-gene-A-BIP	AGA​CGG​CAT​CAT​ATG​GGT​TGC​ACG​GGT​GCC​AAT​GTG​ATC​T	
NEB N-gene-A-LF	GGA​CTG​AGA​TCT​TTC​ATT​TTA​CCG​T	
NEB N-gene-A-LB	ACT​GAG​GGA​GCC​TTG​AAT​ACA	
NEB N2-F3	ACC​AGG​AAC​TAA​TCA​GAC​AAG	Zhang et al. (2020b)
NEB N2-B3	GAC​TTG​ATC​TTT​GAA​ATT​TGG​ATC​T	
NEB N2-FIP	TTC​CGA​AGA​ACG​CTG​AAG​CGG​AAC​TGA​TTA​CAA​ACA​TTG​GCC	
NEB N2-BIP	CGC​ATT​GGC​ATG​GAA​GTC​ACA​ATT​TGA​TGG​CAC​CTG​TGT​A	
NEB N2-LF	GGG​GGC​AAA​TTG​TGC​AAT​TTG	
NEB N2-LB	CTT​CGG​GAA​CGT​GGT​TGA​CC	
DETECTR RNaseP POP7 F3	TTGATGAGCTGGAGCCA	Broughton et al. (2020)
DETECTR RNaseP POP7 B3	CACCCTCAATGCAGAGTC	
DETECTR RNaseP POP7 FIP	GTG​TGA​CCC​TGA​AGA​CTC​GGT​TTT​AGC​CAC​TGA​CTC​GGA​TC	
DETECTR RNaseP POP7 BIP	CCT​CCG​TGA​TAT​GGC​TCT​TCG​TTT​TTT​TCT​TAC​ATG​GCT​CTG​GTC	
DETECTR RNaseP POP7 LF	ATG​TGG​ATG​GCT​GAG​TTG​TT	
DETECTR RNaseP POP7 LB	CAT​GCT​GAG​TAC​TGG​ACC​TC	
ACTB-F3	AGTACCCCATCGAGCACG	Zhang et al. (2020a)
ACTB-B3	AGC​CTG​GAT​AGC​AAC​GTA​CA	
ACTB-FIP	GAG​CCA​CAC​GCA​GCT​CAT​TGT​ATC​ACC​AAC​TGG​GAC​GAC​A	
ACTB-BIP	CTG​AAC​CCC​AAG​GCC​AAC​CGG​CTG​GGG​TGT​TGA​AGG​TC	
ACTB-LoopF	TGT​GGT​GCC​AGA​TTT​TCT​CCA	
ACTB-LoopB	CGA​GAA​GAT​GAC​CCA​GAT​CAT​GT	

**Table:** 

RT-qPCR primers and probes.		
Name	Sequence	References
CDC-N1-F	GAC​CCC​AAA​ATC​AGC​GAA​AT	CDC
CDC-N1-R	TCT​GGT​TAC​TGC​CAG​TTG​AAT​CTG	CDC
CDC-N1-P	FAM-ACCCCGCATTACGTTTGGTGGACC-BHQ1	CDC
CDC-N2-F	TTACAAACATTGGCCGCA AA	CDC
CDC-N2-R	GCGCGACATTCCGAAGAA	CDC
CDC-N2-P	FAM-ACAATTTGCCCCCAGCGCTTCAG-BHQ1	CDC

**Table:** 

Oligos for crRNAs for LbaCas12a		
Name	Sequence	Reference
DETECTR N-gene LbaCas12a guide	GAA​CGC​TGA​AGC​GCT​GGG​GGA​TCT​ACA​CTT​AGT​AGA​AAT​TAc​cct​ata​gtg​agt​cgt​att​aat​ttc	Broughton et al. (2020)
T7-3G IVT primer	GAA​ATT​AAT​ACG​ACT​CAC​TAT​AGG​G	Kellner et al. (2019)

## VCDI Author List

**Stefan Ameres**, Institute of Molecular Biotechnology of the Austrian Academy of Sciences (IMBA), Vienna Biocenter (VBC), Vienna, Austria; **Benedikt Bauer**, Research Institute of Molecular Pathology (IMP), Vienna Biocenter (VBC), Vienna, Austria; **Nikolaus Beer**, Research Institute of Molecular Pathology (IMP), Vienna Biocenter (VBC), Vienna, Austria, Institute of Molecular Biotechnology of the Austrian Academy of Sciences (IMBA), Vienna Biocenter (VBC), Vienna, Austria, and Gregor Mendel Institute (GMI), Austrian Academy of Sciences, Vienna Biocenter (VBC), Vienna, Austria; **Katharina Bergauer**, Research Institute of Molecular Pathology (IMP), Vienna Biocenter (VBC), Vienna, Austria; **Wolfgang Binder**, Max Perutz Labs, Medical University of Vienna, Vienna Biocenter (VBC), Vienna, Austria; **Claudia Blaukopf**, Institute of Molecular Biotechnology of the Austrian Academy of Sciences (IMBA), Vienna Biocenter (VBC), Vienna, Austria; **Boril Bochev**, Research Institute of Molecular Pathology (IMP), Vienna Biocenter (VBC), Vienna, Austria, Institute of Molecular Biotechnology of the Austrian Academy of Sciences (IMBA), Vienna Biocenter (VBC), Vienna, Austria, and Gregor Mendel Institute (GMI), Austrian Academy of Sciences, Vienna Biocenter (VBC), Vienna, Austria; **Julius Brennecke**, Institute of Molecular Biotechnology of the Austrian Academy of Sciences (IMBA), Vienna Biocenter (VBC), Vienna, Austria; **Selina Brinnich**, Vienna Biocenter Core Facilities GmbH (VBCF), Vienna, Austria; **Aleksandra Bundalo**, Research Institute of Molecular Pathology (IMP), Vienna Biocenter (VBC), Vienna, Austria; **Meinrad Busslinger**, Research Institute of Molecular Pathology (IMP), Vienna Biocenter (VBC), Vienna, Austria; **Aleksandr Bykov**, Research Institute of Molecular Pathology (IMP), Vienna Biocenter (VBC), Vienna, Austria; **Tim Clausen**, Research Institute of Molecular Pathology (IMP), Vienna Biocenter (VBC), Vienna, Austria, and Medical University of Vienna, Vienna Biocenter (VBC), Vienna, Austria; **Luisa Cochella**, Research Institute of Molecular Pathology (IMP), Vienna Biocenter (VBC), Vienna, Austria; **Geert de Vries**, Institute of Molecular Biotechnology of the Austrian Academy of Sciences (IMBA), Vienna Biocenter (VBC), Vienna, Austria; **Marcus Dekens**, Research Institute of Molecular Pathology (IMP), Vienna Biocenter (VBC), Vienna, Austria; **David Drechsel**, Research Institute of Molecular Pathology (IMP), Vienna Biocenter (VBC), Vienna, Austria; **Zuzana Dzupinkova**, Research Institute of Molecular Pathology (IMP), Vienna Biocenter (VBC), Vienna, Austria, Institute of Molecular Biotechnology of the Austrian Academy of Sciences (IMBA), Vienna Biocenter (VBC), Vienna, Austria, and Gregor Mendel Institute (GMI), Austrian Academy of Sciences, Vienna Biocenter (VBC), Vienna, Austria; **Michaela Eckmann-Mader**, Vienna Biocenter Core Facilities GmbH (VBCF), Vienna, Austria; **Ulrich Elling**, Institute of Molecular Biotechnology of the Austrian Academy of Sciences (IMBA), Vienna Biocenter (VBC), Vienna, Austria; **Michaela Fellner**, Research Institute of Molecular Pathology (IMP), Vienna Biocenter (VBC), Vienna, Austria; **Thomas Fellner**, Vienna Biocenter Core Facilities GmbH (VBCF), Vienna, Austria; **Laura Fin**, Research Institute of Molecular Pathology (IMP), Vienna Biocenter (VBC), Vienna, Austria; **Bianca Valeria Gapp**, Institute of Molecular Biotechnology of the Austrian Academy of Sciences (IMBA), Vienna Biocenter (VBC), Vienna, Austria; **Gerlinde Grabmann**, Vienna Biocenter Core Facilities GmbH (VBCF), Vienna, Austria; **Irina Grishkovskaya**, Research Institute of Molecular Pathology (IMP), Vienna Biocenter (VBC), Vienna, Austria; **Astrid Hagelkruys**, Institute of Molecular Biotechnology of the Austrian Academy of Sciences (IMBA), Vienna Biocenter (VBC), Vienna, Austria; **Bence Hajdusits**, Research Institute of Molecular Pathology (IMP), Vienna Biocenter (VBC), Vienna, Austria; **David Haselbach**, Research Institute of Molecular Pathology (IMP), Vienna Biocenter (VBC), Vienna, Austria; **Robert Heinen**, Research Institute of Molecular Pathology (IMP), Vienna Biocenter (VBC), Vienna, Austria, Institute of Molecular Biotechnology of the Austrian Academy of Sciences (IMBA), Vienna Biocenter (VBC), Vienna, Austria, and Gregor Mendel Institute (GMI), Austrian Academy of Sciences, Vienna Biocenter (VBC), Vienna, Austria; **Louisa Hill**, Research Institute of Molecular Pathology (IMP), Vienna Biocenter (VBC), Vienna, Austria; **David Hoffmann**, Institute of Molecular Biotechnology of the Austrian Academy of Sciences (IMBA), Vienna Biocenter (VBC), Vienna, Austria; **Stefanie Horer**, Research Institute of Molecular Pathology (IMP), Vienna Biocenter (VBC), Vienna, Austria; **Harald Isemann**, Research Institute of Molecular Pathology (IMP), Vienna Biocenter (VBC), Vienna, Austria; **Robert Kalis**, Research Institute of Molecular Pathology (IMP), Vienna Biocenter (VBC), Vienna, Austria; **Max Kellner**, Research Institute of Molecular Pathology (IMP), Vienna Biocenter (VBC), Vienna, Austria, and Institute of Molecular Biotechnology of the Austrian Academy of Sciences (IMBA), Vienna Biocenter (VBC), Vienna, Austria; **Juliane Kley**, Research Institute of Molecular Pathology (IMP), Vienna Biocenter (VBC), Vienna, Austria; **Thomas Köcher**, Vienna Biocenter Core Facilities GmbH (VBCF), Vienna, Austria; **Alwin Köhler**, Max Perutz Labs, Medical University of Vienna, Vienna Biocenter (VBC), Vienna, Austria; **Darja Kordic**, Research Institute of Molecular Pathology (IMP), Vienna Biocenter (VBC), Vienna, Austria; **Christian Krauditsch**, Institute of Molecular Biotechnology of the Austrian Academy of Sciences (IMBA), Vienna Biocenter (VBC), Vienna, Austria; **Sabina Kula**, Research Institute of Molecular Pathology (IMP), Vienna Biocenter (VBC), Vienna, Austria, Institute of Molecular Biotechnology of the Austrian Academy of Sciences (IMBA), Vienna Biocenter (VBC), Vienna, Austria, and Gregor Mendel Institute (GMI), Austrian Academy of Sciences, Vienna Biocenter (VBC), Vienna, Austria; **Richard Latham**, Research Institute of Molecular Pathology (IMP), Vienna Biocenter (VBC), Vienna, Austria; **Marie-Christin Leitner**, Institute of Molecular Biotechnology of the Austrian Academy of Sciences (IMBA), Vienna Biocenter (VBC), Vienna, Austria; **Thomas Leonard**, Max Perutz Labs, Medical University of Vienna, Vienna Biocenter (VBC), Vienna, Austria; **Dominik Lindenhofer**, Institute of Molecular Biotechnology of the Austrian Academy of Sciences (IMBA), Vienna Biocenter (VBC), Vienna, Austria; **Raphael Arthur Manzenreither**, Institute of Molecular Biotechnology of the Austrian Academy of Sciences (IMBA), Vienna Biocenter (VBC), Vienna, Austria; **Karl Mechtler**, Research Institute of Molecular Pathology (IMP), Vienna Biocenter (VBC), Vienna, Austria; **Anton Meinhart**, Research Institute of Molecular Pathology (IMP), Vienna Biocenter (VBC), Vienna, Austria; **Stefan Mereiter**, Institute of Molecular Biotechnology of the Austrian Academy of Sciences (IMBA), Vienna Biocenter (VBC), Vienna, Austria; **Thomas Micheler**, Vienna Biocenter Core Facilities GmbH (VBCF), Vienna, Austria; **Paul Moeseneder**, Institute of Molecular Biotechnology of the Austrian Academy of Sciences (IMBA), Vienna Biocenter (VBC), Vienna, Austria; **Tobias Neumann**, Research Institute of Molecular Pathology (IMP), Vienna Biocenter (VBC), Vienna, Austria; **Simon Nimpf**, Research Institute of Molecular Pathology (IMP), Vienna Biocenter (VBC), Vienna, Austria; **Magnus Nordborg**, Gregor Mendel Institute (GMI), Austrian Academy of Sciences, Vienna Biocenter (VBC), Vienna, Austria; **Egon Ogris**, Max Perutz Labs, Medical University of Vienna, Vienna Biocenter (VBC), Vienna, Austria; **Michaela Pagani**, Research Institute of Molecular Pathology (IMP), Vienna Biocenter (VBC), Vienna, Austria; **Andrea Pauli**, Research Institute of Molecular Pathology (IMP), Vienna Biocenter (VBC), Vienna, Austria; **Jan-Michael Peters**, Research Institute of Molecular Pathology (IMP), Vienna Biocenter (VBC), Vienna, Austria, and Medical University of Vienna, Vienna Biocenter (VBC), Vienna, Austria; **Petra Pjevac**, Centre for Microbiology and Environmental Systems Science, University of Vienna, Vienna, Austria, and Joint Microbiome Facility of the University of Vienna and Medical University of Vienna, Vienna, Austria; **Clemens Plaschka**, Research Institute of Molecular Pathology (IMP), Vienna Biocenter (VBC), Vienna, Austria; **Martina Rath**, Research Institute of Molecular Pathology (IMP), Vienna Biocenter (VBC), Vienna, Austria; **Daniel Reumann**, Institute of Molecular Biotechnology of the Austrian Academy of Sciences (IMBA), Vienna Biocenter (VBC), Vienna, Austria; **Sarah Rieser**, Research Institute of Molecular Pathology (IMP), Vienna Biocenter (VBC), Vienna, Austria; **Marianne Rocha-Hasler**, Centre for Microbiology and Environmental Systems Science, University of Vienna, Vienna, Austria; **Alan Rodriguez**, Research Institute of Molecular Pathology (IMP), Vienna Biocenter (VBC), Vienna, Austria, and Institute of Molecular Biotechnology of the Austrian Academy of Sciences (IMBA), Vienna Biocenter (VBC), Vienna, Austria; **James Julian Ross**, Institute of Molecular Biotechnology of the Austrian Academy of Sciences (IMBA), Vienna Biocenter (VBC), Vienna, Austria; **Harald Scheuch**, Research Institute of Molecular Pathology (IMP), Vienna Biocenter (VBC), Vienna, Austria, Institute of Molecular Biotechnology of the Austrian Academy of Sciences (IMBA), Vienna Biocenter (VBC), Vienna, Austria, and Gregor Mendel Institute (GMI), Austrian Academy of Sciences, Vienna Biocenter (VBC), Vienna, Austria; **Karina Schindler**, Research Institute of Molecular Pathology (IMP), Vienna Biocenter (VBC), Vienna, Austria; **Clara Schmidt**, Institute of Molecular Biotechnology of the Austrian Academy of Sciences (IMBA), Vienna Biocenter (VBC), Vienna, Austria; **Hannes Schmidt**, Centre for Microbiology and Environmental Systems Science, University of Vienna, Vienna, Austria; **Jakob Schnabl**, Institute of Molecular Biotechnology of the Austrian Academy of Sciences (IMBA), Vienna Biocenter (VBC), Vienna, Austria; **Stefan Schüchner**, Max Perutz Labs, Medical University of Vienna, Vienna Biocenter (VBC), Vienna, Austria; **Tanja Schwickert**, Research Institute of Molecular Pathology (IMP), Vienna Biocenter (VBC), Vienna, Austria; **Andreas Sommer**, Vienna Biocenter Core Facilities GmbH (VBCF), Vienna, Austria; **Johannes Stadlmann**, Institute of Biochemistry, University of Natural Resources and Life Sciences (BOKU), Vienna, Austria; **Alexander Stark**, Research Institute of Molecular Pathology (IMP), Vienna Biocenter (VBC), Vienna, Austria, and Medical University of Vienna, Vienna Biocenter (VBC), Vienna, Austria; **Peter Steinlein**, Research Institute of Molecular Pathology (IMP), Vienna Biocenter (VBC), Vienna, Austria, Institute of Molecular Biotechnology of the Austrian Academy of Sciences (IMBA), Vienna Biocenter (VBC), Vienna, Austria, and Gregor Mendel Institute (GMI), Austrian Academy of Sciences, Vienna Biocenter (VBC), Vienna, Austria; **Simon Strobl**, Vienna Biocenter Core Facilities GmbH (VBCF), Vienna, Austria; **Qiong Sun**, Research Institute of Molecular Pathology (IMP), Vienna Biocenter (VBC), Vienna, Austria; **Wen Tang**, Research Institute of Molecular Pathology (IMP), Vienna Biocenter (VBC), Vienna, Austria; **Linda Trübestein**, Max Perutz Labs, Medical University of Vienna, Vienna Biocenter (VBC), Vienna, Austria; **Christian Umkehrer**, Research Institute of Molecular Pathology (IMP), Vienna Biocenter (VBC), Vienna, Austria; **Sandor Urmosi-Incze**, Vienna Biocenter Core Facilities GmbH (VBCF), Vienna, Austria; **Kristina Uzunova**, Research Institute of Molecular Pathology (IMP), Vienna Biocenter (VBC), Vienna, Austria, Institute of Molecular Biotechnology of the Austrian Academy of Sciences (IMBA), Vienna Biocenter (VBC), Vienna, Austria, and Gregor Mendel Institute (GMI), Austrian Academy of Sciences, Vienna Biocenter (VBC), Vienna, Austria; **Gijs Versteeg**, Department of Microbiology, Immunobiology, and Genetics, Max Perutz Labs, University of Vienna, Vienna Biocenter (VBC), Dr. Bohr-Gasse 9, 1030 Vienna, Austria; **Alexander Vogt**, Vienna Biocenter Core Facilities GmbH (VBCF), Vienna, Austria; **Vivien Vogt**, Research Institute of Molecular Pathology (IMP), Vienna Biocenter (VBC), Vienna, Austria; **Michael Wagner**, Centre for Microbiology and Environmental Systems Science, University of Vienna, Vienna, Austria, and Joint Microbiome Facility of the University of Vienna and Medical University of Vienna, Vienna, Austria; **Martina Weissenboeck**, Research Institute of Molecular Pathology (IMP), Vienna Biocenter (VBC), Vienna, Austria; **Barbara Werner**, Vienna Biocenter Core Facilities GmbH (VBCF), Vienna, Austria; **Ramesh Yelagandula**, Institute of Molecular Biotechnology of the Austrian Academy of Sciences (IMBA), Vienna Biocenter (VBC), Vienna, Austria; **Johannes Zuber**, Research Institute of Molecular Pathology (IMP), Vienna Biocenter (VBC), Vienna, Austria, and Medical University of Vienna, Vienna Biocenter (VBC), Vienna, Austria.

## Data Availability

The original contributions presented in the study are included in the article/Supplementary Material, further inquiries can be directed to the corresponding authors.
