# Supplementary material for: A Rapid, Highly Sensitive and Open-Access SARS-CoV-2 Detection Assay for Laboratory and Home Testing
Source: Front Mol Biosci. 2022 Apr 1;9:801309. doi: 10.3389/fmolb.2022.801309 (PMC9011764; doi:10.3389/fmolb.2022.801309)
Supplement: Supplementary file 1 [file DataSheet1.DOCX]

Supplementary Material

# Supplementary Figures

**Figure S1: Primer performance for the detection of SARS-CoV-2 by RT-LAMP.**

**A)** Amplification curves (real-time fluorescent measurements; in duplicates) from RT-LAMP reactions shown in Figure 1C. Curves using synthetic SARS-CoV-2 RNA standard dilutions as input are in color (color-code indicates different primer sets; yellow: As1 Orf1ab; red: E-gene E1 NEB; blue: N-gene N2 DETECTR). Curves using non-targeting controls (NTC) as input are shown in black. **B)** Dot-plot showing time to threshold (in minutes) of RT-LAMP reactions using As1 or NEB E1 Primer on a dilution series containing various amounts of synthetic Twist SARS-CoV-2 RNA (12 replicates per condition). The co-measured RT-qPCR Ct value for each dilution as well as the number of RT-LAMP positive reactions are indicated below. Reactions not positive after 35 minutes are considered ‘not detected’. **C)** End-point fluorescence measured for RT-LAMP reactions shown in B) after 35 minutes**. D)** Reaction sensitivity, expressed as % Positive, obtained for reactions containing synthetic Twist SARS-CoV-2 and As1 or NEB E1 Primer**.** Dilutions were grouped into bins, for which sample proportions and 95% confidence limits (Wilson/Brown Method) were calculated and plotted with error bars representing upper and lower intervals. **E)** Heatmap showing end-point relative fluorescence values (after 35 minutes) of RT-LAMP reactions (in duplicates; respective primers indicated to the left) using COVID-19 patient samples with indicated Cq values (determined via RT-qPCR and the N1-CDC amplicon) as input. Reactions with primers targeting the human ACTB gene served as sample quality control. All reactions were performed in duplicates.


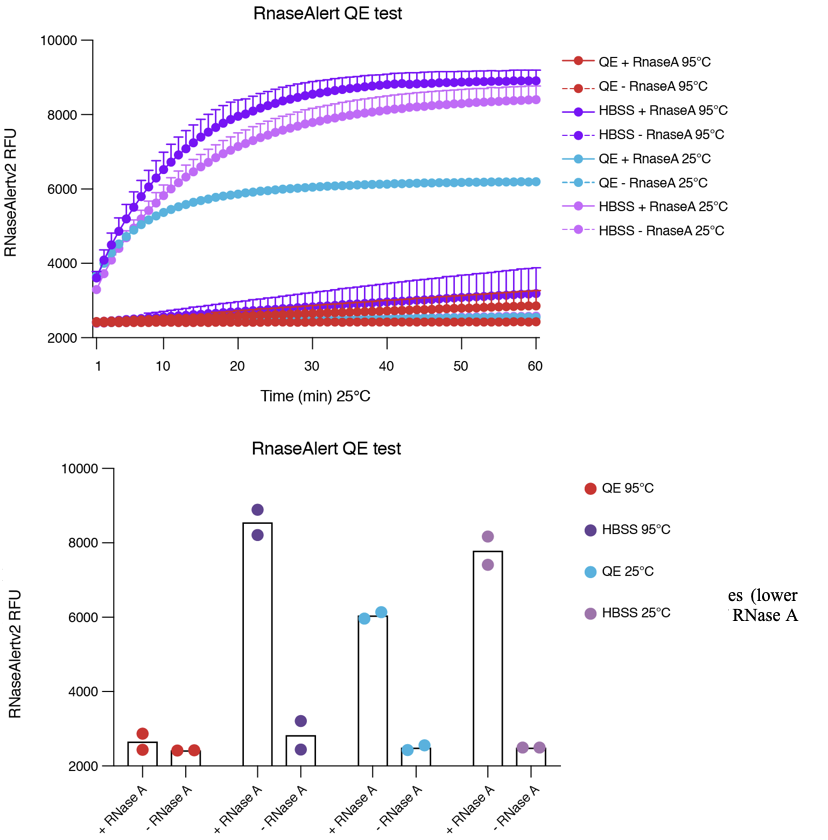


**Figure S2: QuickExtract buffer combined with heat inhibits RNase activity.**

Shown are relative fluorescence values over time (upper graph) and end-point fluorescence values (lower graph) of RNaseAlert reactions in HBSS buffer or 1x QuickExtract, in the presence or absence of RNase A and with or without incubation at 95°C. All reactions were performed in duplicates. The experimental workflow was as follows: 1) Different solutions (HBSS, or QuickExtract) were supplemented with RNase A (control no RNase A); 2) Reactions were incubated at room temperature or 95°C for 5 min, followed by 3) addition of a quenched fluorophore RNA reporter (RNaseAlert) and incubation at room temperature for 1 hour, during which fluorescence was recorded at 1-minute intervals.


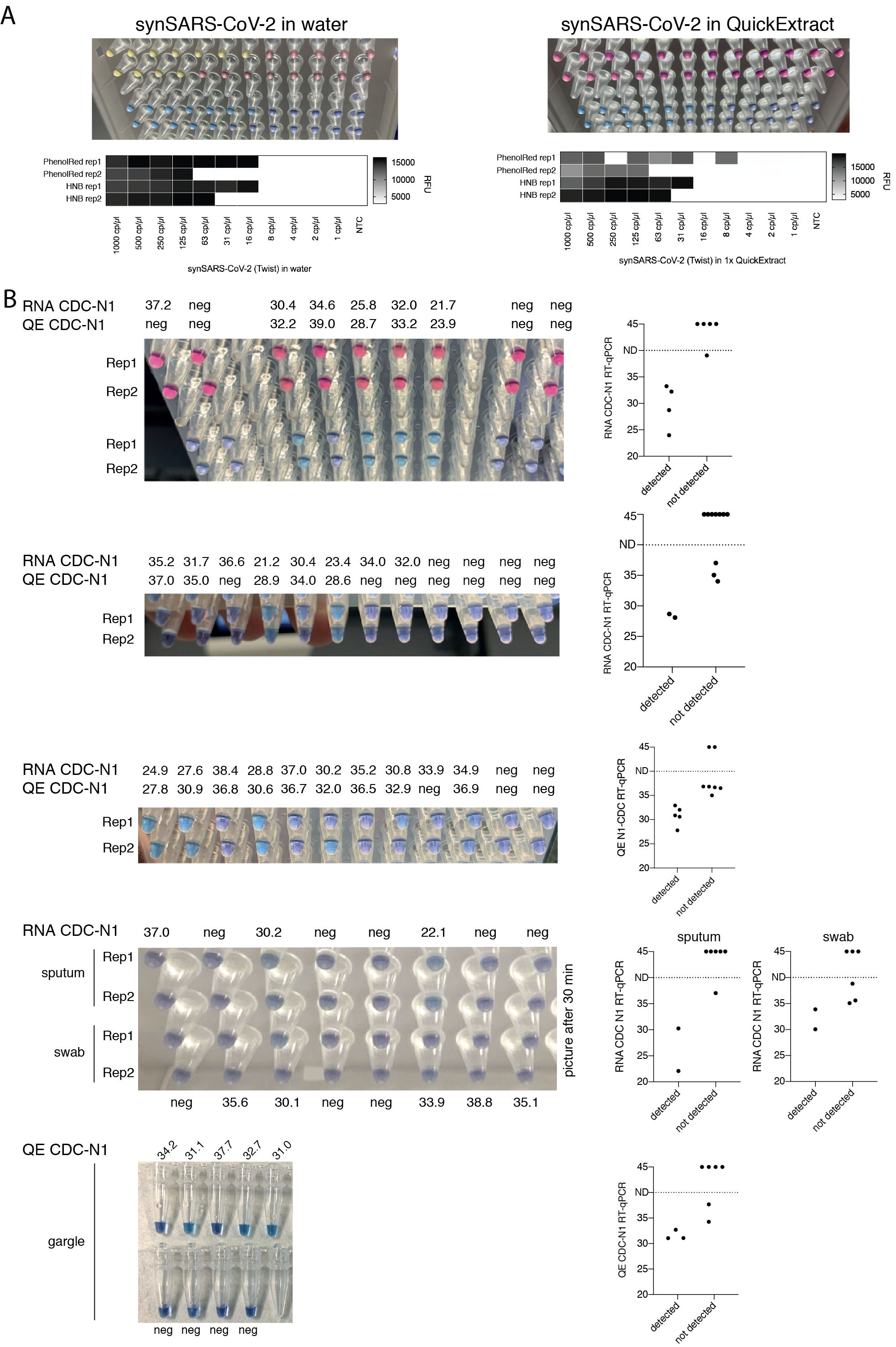


**Figure S3: HNB RT-LAMP shows robust performance on QuickExtract-treated patient sample material.**

**A)** Comparison between Phenol Red and HNB colorimetric readout of RT-LAMP reactions (in duplicates) on serially diluted synthetic SARS-CoV-2 RNA in water (left) or 1x QuickExtract (right). End-point fluorescence values measured in parallel are shown in heatmaps below. While fluorescent detection indicates successful LAMP in both sample matrices, Phenol Red but not HNB colorimetric readout is inconclusive in QuickExtract buffer (right panel, top rows). **B)** HNB RT-LAMP performance across a wide range of COVID-19 patients and sample types. Images showing the HNB end-point outcome of RT-LAMP reactions on multiple COVID-19 patient samples (gargle, swab or sputum; samples indicate swabs if not otherwise stated). The respective Cq values of the individual samples (CDC-N1; QuickExtract RT-qPCR or extracted RNA RT-qPCR as indicated) are shown above or below each sample. Colorimetric RT-LAMP using Phenol Red is shown for one sample set (first from top), again with inconclusive outcome. All reactions were performed in duplicates. The HNB color-reaction was read-out at 35 minutes unless indicated otherwise. Summary dotplots for every sample set are shown to the right; samples were classified as detected or not detected based on RT-LAMP outcome and plotted against their respective RT-qPCR determined Cq values.

**
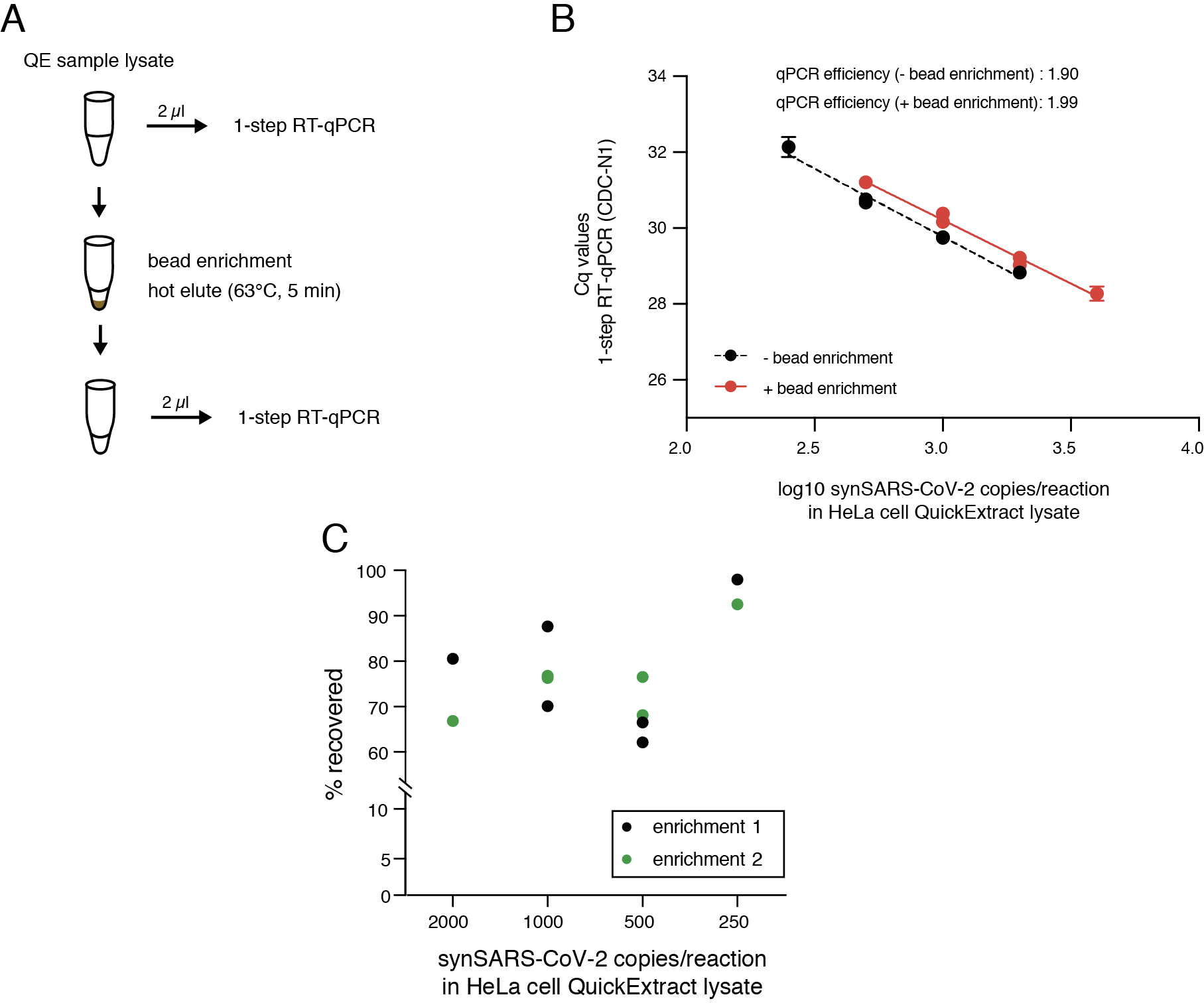
**

**Figure S4: Assessment of the bead enrichment procedure.**

**A)** Schematic depicting the workflow to assess bead recovery performance. Synthetic SARS-CoV-2 standard was diluted in HBSS:QuickExtract lysis buffer (1:1). 40 µl were subjected to magnetic bead enrichment, followed by elution of nucleic acids in 20 µl of RNase-free water by incubation at 63˚C for 5 min. 2 µl of the input (before enrichment) and the eluate (after bead enrichment) were analysed by 1-step RT-qPCR. **B)** RT-qPCR Cq values of different dilutions of synthetic SARS-CoV-2 standard before (black) and after bead enrichment (red). The mean Cq value and standard deviation from two technical replicates are shown. The x-axis indicates the theoretical sample concentration before (black) and after bead-enrichment (red), which increases 2-fold after enrichment. The qPCR efficiency was calculated as E = -1+10(-1/slope) of the linear regression of datapoints. **C)** Calculated recovery (in %) after bead enrichment for the dilution series of synthetic SARS-CoV-2 RNA standard measured in B) is shown. Two independent bead enrichment experiments were performed and are shown in black and green. The recovery rate was calculated from RT-qPCR measured sample concentrations before and after enrichment, while also considering the respective enrichment factor.


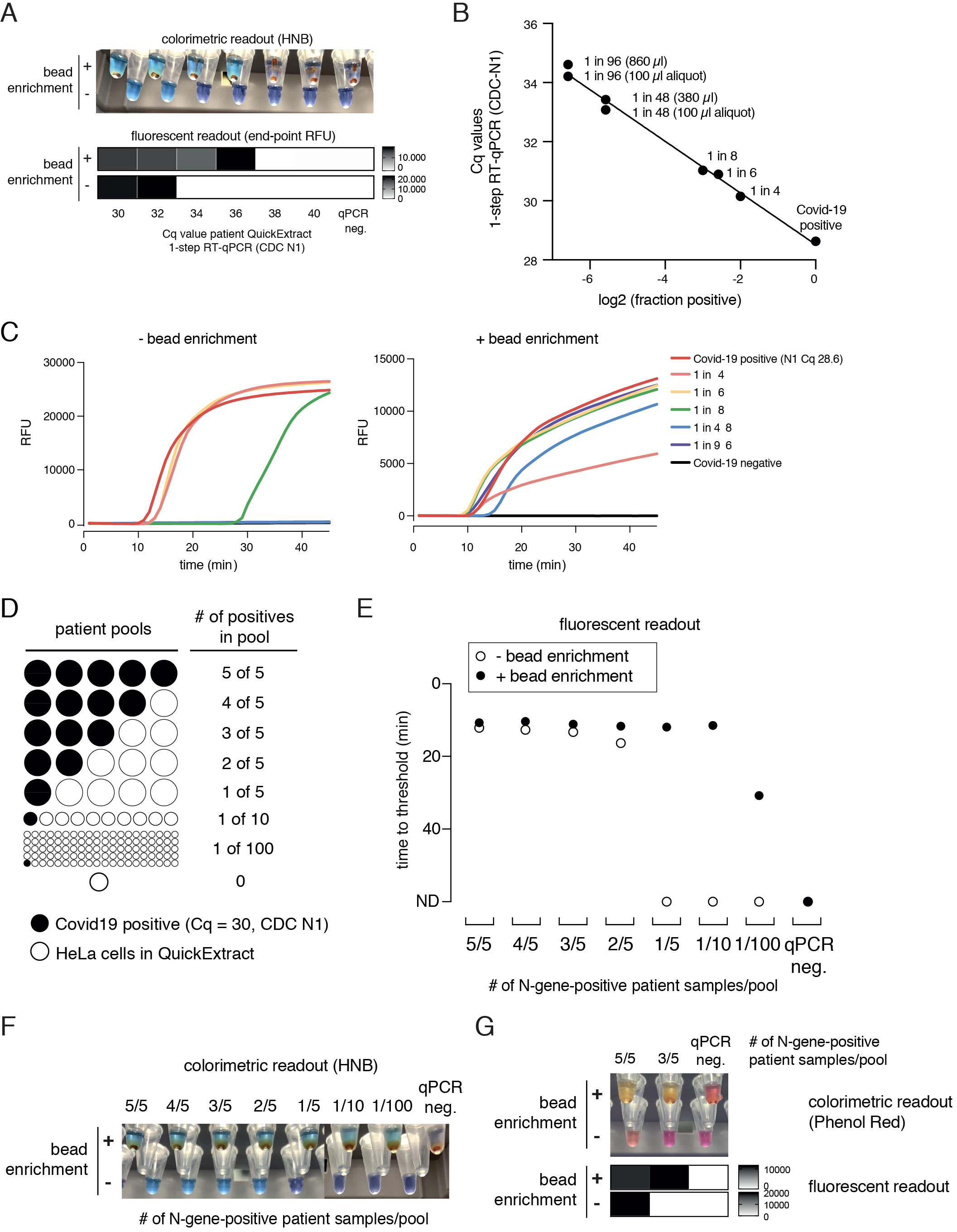


**Figure S5: Pooled COVID-19 testing strategy using bead-LAMP.**

**A)** Performance of bead-LAMP on crude patient samples. The image (top) shows HNB end-point colorimetric readout and the heatmap (bottom) shows co-measured end-point relative fluorescence units (RFUs) of RT-LAMP on serially diluted patient samples in QuickExtract-prepared HeLa cell lysate, with or without prior bead enrichment. Cq values are estimates based on dilution of an undiluted and quantified COVID-19 patient sample prior to bead enrichment. All reactions were performed in duplicates. **B)** RT-qPCR Cq values (CDC-N1) of gargle sample pools used in Fig 4H-J with the indicated fraction of COVID-19 positive gargle sample per pool. For the two large sample pools (pool of 1 in 96 and pool of 1 in 48), the 100 µl aliquot used for subsequent RT-LAMP and bead-LAMP was measured in addition. **C)** Readout of a real-time fluorescence RT-LAMP reaction of sample pools with indicated fraction of positive lysate without (left) and with (right) bead enrichment. RFU: relative fluorescent units. **D)** Schematic illustrating the pooled testing strategy. Eight pools mimicking different total patient sample numbers and different ratios of COVID-19-positive patient samples (0-100%) were generated from one COVID-19-positive QuickExtract patient sample (N1 RT-qPCR with Cq ~30) mixed at the indicated ratios with QuickExtract HeLa cell lysate at 20 cells/µl. **E)** Shown is the performance (measured as end-point relative fluorescence units (RFU)) of bead-LAMP (filled circles) compared to regular RT-LAMP (open circles) on the patient pools defined in D. ND = not detected within 60 minutes of RT-LAMP incubation. **F)** Images showing the endpoint HNB colorimetric readout of samples measured in E with or without prior bead enrichment. **G)** Bead-enrichment makes crude QuickExtract samples compatible with the pH-sensitive Phenol Red based colorimetric readout of RT-LAMP. Images showing the endpoint Phenol Red colorimetric readout (top) and the fluorescent readout (bottom) of two COVID-19 positive pools and one COVID-19 negative pool (qPCR negative) defined in D with (+) or without (-) prior bead enrichment.

**
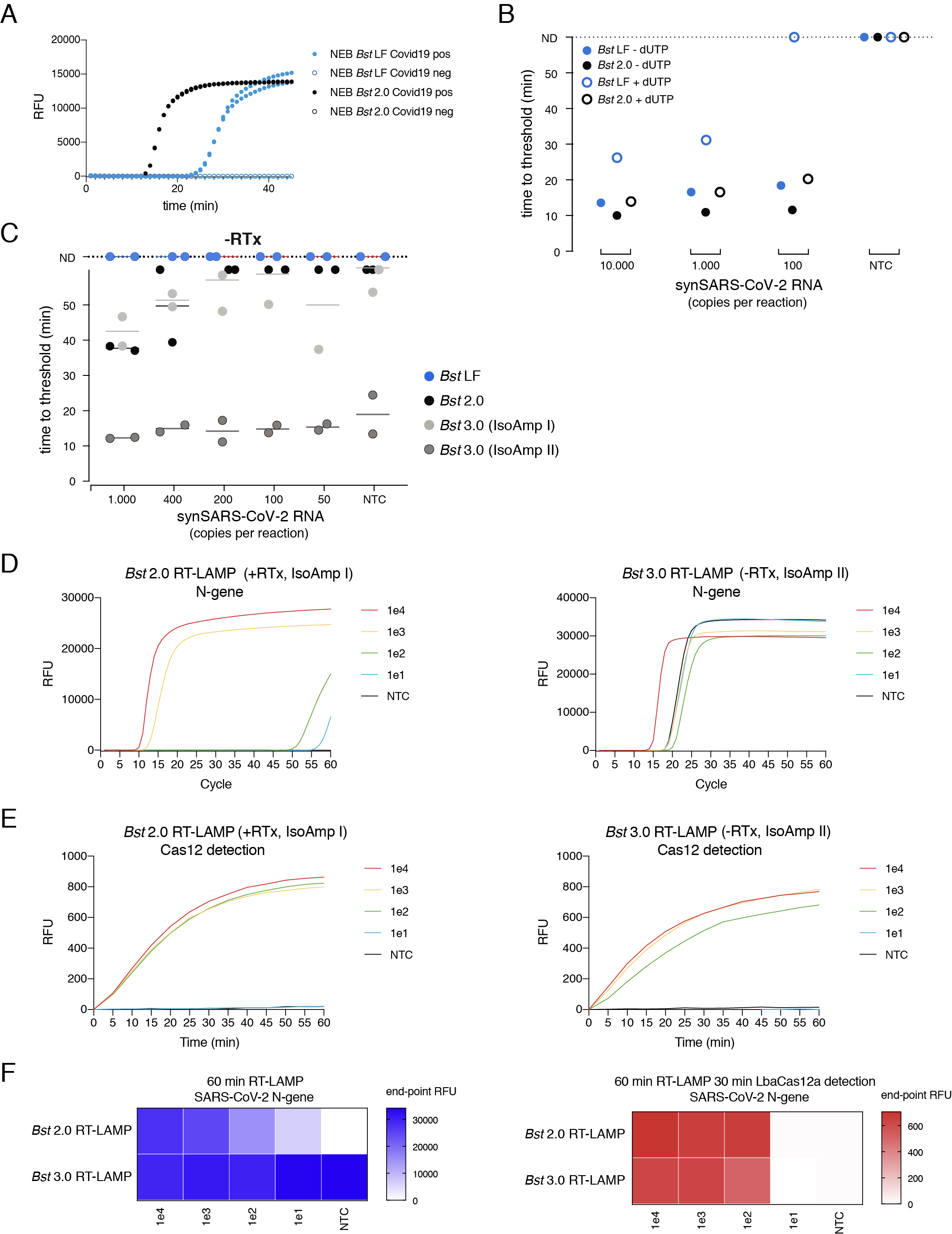
**

**Figure S6: Comparison of different *Bst* polymerases for RT-LAMP.**

**A)** Performance of *Bst* LF (blue curves) or *Bst* 2.0 (black curves) on crude COVID-19 patient sample (prepared in QuickExtract). Amplification curves indicate real-time fluorescence measurements of RT-LAMP reactions (E1 primer set; in duplicates) using SARS-CoV-2 positive (filled circles) or SARS-CoV-2 negative (open circles) patient samples as input. **B)** Comparison of the ability of wildtype (*Bst* LF, blue) and engineered *Bst* polymerase (*Bst* 2.0, black) to incorporate dUTP during RT-LAMP on synthetic SARS-CoV-2 RNA standard. Reactions were either run as self-assembled RT-LAMP reactions under standard conditions (-dUTP, filled circles), or supplemented with 0.7 mM dUTP, 0.7 mM dTTP and 1.4 mM of each dATP, dCTP, dGTP (open circles). Plotted is the ‘time to threshold’ as a measure of performance. **C)** LAMP performance (given as time to threshold in minutes) of indicated *Bst* DNA polymerase variants in the absence of a dedicated reverse transcriptase (-RTx) using diluted synthetic SARS-CoV-2 RNA (copies per reaction indicated) as template (related to Figure 6A). **D)** RT-LAMP real-time fluorescence measurements using RTx and *Bst* 2.0 in IsoAmp buffer I (left) versus *Bst* 3.0 alone in IsoAmp buffer II (right). N2 DETECTR was used as primer set for amplifying synthetic SARS-CoV-2 RNA standard (copy number per reaction is indicated; no target control (NTC): water). **E)** Shown is the collateral cleavage activity (measured as real-time fluorescent signal) by Cas12, with a crRNA targeting the N2 LAMP amplicon, upon addition of 2 µl of LAMP reactions from D) to 20 µl of Cas12 cleavage mix. **F)** (Left) End-point fluorescence values (after 60 minutes) of RT-LAMP reactions from D). (Right) Cas12-based detection of LAMP products from D) is indicated.

**
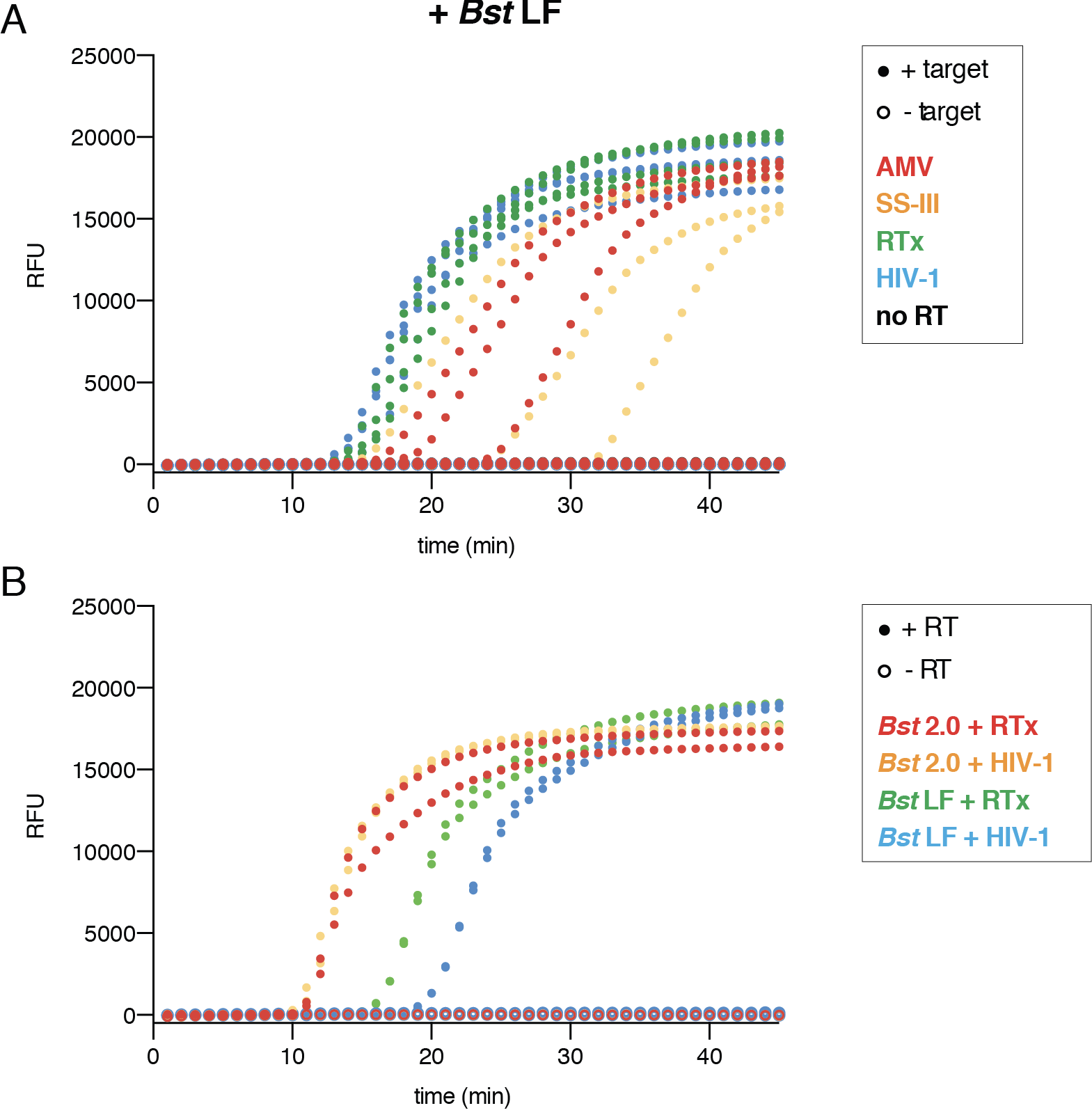
**

**Figure S7: Comparison of different reverse transcriptases and *Bst* polymerases for RT-LAMP.**

**A)** Amplification curves (real-time fluorescent measurements; in triplicates) from RT-LAMP reactions shown in Figure 6B. **B)** Amplification curves (real-time fluorescent measurements; in duplicates) from RT-LAMP reactions shown in Figure 6D.
